# How do clinicians use post-COVID syndrome diagnosis? Analysis of clinical features in a Swedish COVID-19 cohort with 18 months’ follow-up: a national observational cohort and matched cohort study

**DOI:** 10.1136/bmjph-2023-000336

**Published:** 2024-03-25

**Authors:** Hanna M Ollila, Osvaldo Fonseca-Rodríguez, Ida Henriette Caspersen, Sebastian Kalucza, Johan Normark, Lill Trogstad, Per Minor Magnus, Naja Hulvej Rod, Andrea Ganna, Marie Eriksson, Anne-Marie Fors Connolly

**Affiliations:** 1 Institute for Molecular Medicine, FIMM, University of Helsinki, Helsinki, Finland; 2 Massachusetts General Hospital Center for Genomic Medicine, Boston, Massachusetts, USA; 3 Department of Anesthesia, Critical Care and Pain Medicine, Massachusetts General Hospital and Harvard Medical School, Boston, Massachusetts, USA; 4 Broad Institute, Cambridge, Massachusetts, USA; 5 Department of Clinical Microbiology, Umeå University, Umeå, Sweden; 6 Centre for Fertility and Health, Norwegian Institute of Public Health, Oslo, Norway; 7 Department of Public Health, University of Copenhagen, Copenhagen, Denmark; 8 Department of Statistics, Umeå University, Umeå, Sweden

**Keywords:** COVID-19, epidemiology, public health

## Abstract

**Introduction:**

SARS-CoV-2 infection causes acute COVID-19 and may result in post-COVID syndrome (PCS). We aimed to investigate how clinicians diagnose PCS and identify associated clinical and demographic characteristics.

**Methods:**

We analysed multiregistry data of all SARS-CoV-2 test-positive individuals in Sweden (n=1 057 174) between 1 February 2020 and 25 May 2021. We described clinical characteristics that prompt PCS diagnosis in outpatient and inpatient settings. In total, there were 6389 individuals with a hospital inpatient or outpatient diagnosis for PCS. To understand symptomatology, we examined individuals diagnosed with PCS at least 3 months after COVID-19 onset (n=6389) and assessed factors associated with PCS diagnosis.

**Results:**

Mechanical ventilation correlated with PCS (OR 114.7, 95% CI 105.1 to 125.3) compared with no outpatient/inpatient contact during initial COVID-19. Dyspnoea (13.4%), malaise/fatigue (8%) and abnormal pulmonary diagnostic imaging findings (4.3%) were the most common features linked to PCS. We compared clinical features of PCS with matched controls (COVID-19 negative, n=23 795) and COVID-19 severity-matched patients (COVID-19 positive, n=25 556). Hypertension associated with PCS cohort (26.61%) than in COVID-19-negative (OR 17.16, 95% CI 15.23 to 19.3) and COVID-19-positive (OR 9.25, 95% CI 8.41 to 10.16) controls, although most individuals received this diagnosis before COVID-19. Dyspnoea was the second most common feature in the PCS cohort (17.2%), and new to the majority compared with COVID-19-negative (OR 54.16, 95% CI 42.86 to 68.45) and COVID-19-positive (OR 18.7, 95% CI 16.21 to 21.57) controls.

**Conclusions:**

Our findings highlight factors Swedish physicians associate with PCS.

**WHAT IS ALREADY KNOWN ON THIS TOPIC**

**WHAT THIS STUDY ADDS**

- This study, based on the total population of laboratory-verified SARS-CoV-2-infected individuals with data from multiple nationwide registries in Sweden, reveals that mechanical ventilation during initial COVID-19 treatment is strongly associated with PCS. The most common clinical features linked to PCS are dyspnoea, malaise/fatigue and abnormal pulmonary diagnostic imaging findings. Hypertension and dyspnoea were found to be more common in the PCS cohort than in both COVID-19-negative and COVID-19-positive controls, though the majority had been diagnosed with hypertension previously. Dyspnoea was new to the majority of PCS individuals.

**HOW THIS STUDY MIGHT AFFECT RESEARCH, PRACTICE OR POLICY**

- These findings provide valuable insights for clinicians diagnosing PCS and could inform future research into its causes and treatment. They may also influence policy decisions related to the care and management of patients recovering from COVID-19.

## Introduction

 Infection with SARS-CoV-2 resulting in COVID-19 exhibits a wide range of symptoms including respiratory and pulmonary symptoms during the acute phase of disease.^1^,^3^ The key characteristics include remarkably variable individual health responses from those who are asymptomatic or have mild symptoms to those with life-threatening severe disease or even death.^1 2^ An increasing number of patients suffer from disabling long-term consequences, post-COVID syndrome (PCS, also known as Long COVID, post-acute sequelae of COVID-19 or post-acute COVID-19 syndrome). Furthermore, the symptomatology of PCS varies between patients creating a challenge both for disease diagnosis, management and public health overview of the extent of the problem.

The global consensus on how PCS should be diagnosed and treated is still developing based on the efforts from the WHO, patient organisations and the Centre of Disease Control in providing guidelines and International Classification of Diseases (ICD) codes for diagnosis. PCS is thought to affect multiple organs,^4^,^6^ and potentially initial severity of COVID-19 could be a risk factor for PCS.^6^,^8^ Our understanding of the magnitude of how much vaccines protect against PCS is evolving.^9 10^ Breakthrough infections following vaccination and reinfection with SARS-CoV-2 also associate with long-term organ sequelae.^9 11^

The symptoms attributed to PCS are still being characterised, and there could be several different clinical phenotypes; however, dyspnoea and fatigue seem to be central symptomatic components of PCS.^56 12^,^15^ Post-intensive care syndrome (PICS) refers to a set of physical, cognitive and psychological impairments that survivors of intensive care often experience, with issues like muscle weakness, memory loss and depression.^16^ This could be similar to issues found in PCS.

To understand how clinicians attribute symptoms and PCS diagnosis, we examined risk factors and associated clinical features in patients who received a PCS ICD code in a total population of COVID-19 cohort in Sweden. To avoid classifying symptomatology due to PICS, COVID-19 patients with PCS were compared with disease severity-matched COVID-19 patients with no PCS diagnosis.

## Methods

### Definition of PCS

In September 2020, the WHO introduced ICD 10th version (ICD-10) code (U08.9 and U09.9) for PCS to distinguish between acute and late effects of COVID-19 infection. In Sweden, Z86.1A was implemented from 1 June 2020 to identify individuals with ‘COVID-19 in the personal history’, but was discontinued from 1 January 2021.^17^ The definition of post-COVID-19 condition by the WHO was: ‘Post-COVID-19 condition occurs in individuals with a history of probable or confirmed SARS-CoV-2 infection, usually 3 months from the onset of COVID-19 with symptoms that last for at least 2 months and cannot be explained by an alternative diagnosis’.^18^ Based on this definition, we defined PCS as the presence of the ICD-10 diagnostic codes Z86.1A, U08.9 and U09.9 that occurred at least 3 months post-COVID-19 in visits to outpatient and inpatient clinics (online supplemental file 1).

### Source of data and study population

COVID-19 is a notifiable disease in Sweden and all laboratory-verified test-positive individuals are registered in the centralised communicable disease surveillance network (SmiNet) administered by the Swedish Public Health Agency, thereby including the spectrum of COVID-19 severity from those who do not display symptoms to those with fatal COVID-19, and has been described in previous studies.^19^,^22^ The personal identity numbers (PINs) from individuals with a laboratory-verified test-positive SARS-CoV-2 infection were extracted from SmiNet, and cross-linked with the following nationwide registries: Longitudinal Integrated Database for Health Insurance and Labour Market Studies registry (socioeconomic variables); Patient, Cancer, Death and Intensive Care Registries that are administered by Statistics Sweden, the Swedish National Board of Health and Welfare and the Swedish Intensive Care registry, respectively. Only COVID-19 patients who survived until at least 3 months after COVID-19 were included. Primary care data are not registered centrally and are therefore not accessible to perform a national study.

To identify clinical features clinicians associate with PCS (using diagnoses as proxy) and account for diseases prevalent in the background population/COVID-19 cohort not interpreted to be related to PCS by Swedish physicians in an outpatient/inpatient setting, we compared ICD-10 codes registered at healthcare visits at least 3 months after COVID-19 regardless of cause of visit for individuals with PCS; with ICD-10 codes registered at healthcare visits 3 months after index date or COVID-19 date for four control individuals matched on age, sex and county of residence that were either COVID-19 negative (no report of laboratory-verified positive SARS-CoV-2 test in SmiNet) or COVID-19 positive. The COVID-19-positive control individuals were also matched according to disease severity to ensure PICS did not factor into our study as a potential explanation for clinical features of PCS. The index date for COVID-19-negative control individuals was the corresponding COVID-19 date for the matched case, and they were identified by Statistics Sweden, and the four COVID-19-positive control individuals were identified from the COVID-19 cohort using R V.4.1.0 (R Core Team, 2021).

The data were pseudonymised by converting all PINs to a study ID.

### Factors associating with receiving a PCS diagnosis by Swedish physicians

Potential factors under study included sex, age, disease severity, education, income and comorbidities. Age was grouped into 0–19, 20–39, 40–59, 60–79 and 80+ years. Disease severity was categorised into ‘no contact with outpatient or inpatient clinics’, ‘contact with outpatient clinic’, ‘hospitalisation’, ‘non-invasive ventilation and high-flow oxygen’, ‘intensive care’ and ‘mechanical ventilation’. Highest level of education was defined as primary, secondary or tertiary school and annual disposable income was categorised into quintiles. Comorbidities were summarised by the weighted Charlson Comorbidity Index (wCCI) and calculated using the algorithm designed specifically for Swedish registries,^23^ and based on ICD codes registered in the Patient and Cancer Registries from 1 January 1997 up to 1 month before the COVID-19/index date.

### Clinical features physicians associate with PCS

The clinic and geographical region were characterised for the outpatient and inpatient clinics that recorded PCS diagnoses. Associated ICD-10 codes registered at PCS visits were used as a proxy to describe clinical features of PCS, and only ICD-10 codes where at least 1% of the cohort received these are included. The ICD-10 Z-chapter (factors influencing health status and contact with health services) was excluded from all analyses. Whether the diagnoses were new registrations or registered prior to COVID-19 was determined by searching historically for all visits to healthcare from 1 January 1997 to 1 month prior to COVID-19 (online supplemental file 1).

### Statistical analysis

Potential factors associating with receiving a PCS diagnosis (sex, age, education, income, wCCI and CCI groups) were presented by descriptive statistics. The association between risk factors and PCS in the nationwide COVID-19 cohort was analysed using logistic regression adding variables in a stepwise process. Model 0 was a univariable model; model 1 included disease severity, age and sex; followed by addition of wCCI (model 2); addition of education (model 3); addition of income but not education (model 4) and finally addition of all variables (model 5). To identify which comorbidity groups were associated with PCS, a model including all variables (model 5) and individual CCI comorbidity groups were included.

Conditional logistic regression was used to identify diagnostic codes that associate with PCS compared with the matched COVID-19-negative and COVID-19-positive control individuals. The original p values were adjusted by false discovery rate and associations below 5% were considered significant. Outcomes from logistic regression models were presented with ORs and 95% CIs. Outcomes with p<0.05 were considered statistically significant. Missing data were included as a category of their own. Statistical analyses were carried out using R V.4.1.0 (R Core Team, 2021).

### Patient and public involvement

The manuscript was circulated among a Swedish PCS patient group comprising of physicians and the Patient-Led Research Collaborative for comments and feedback, which were incorporated into the manuscript.

## Results

**Figure 1:**
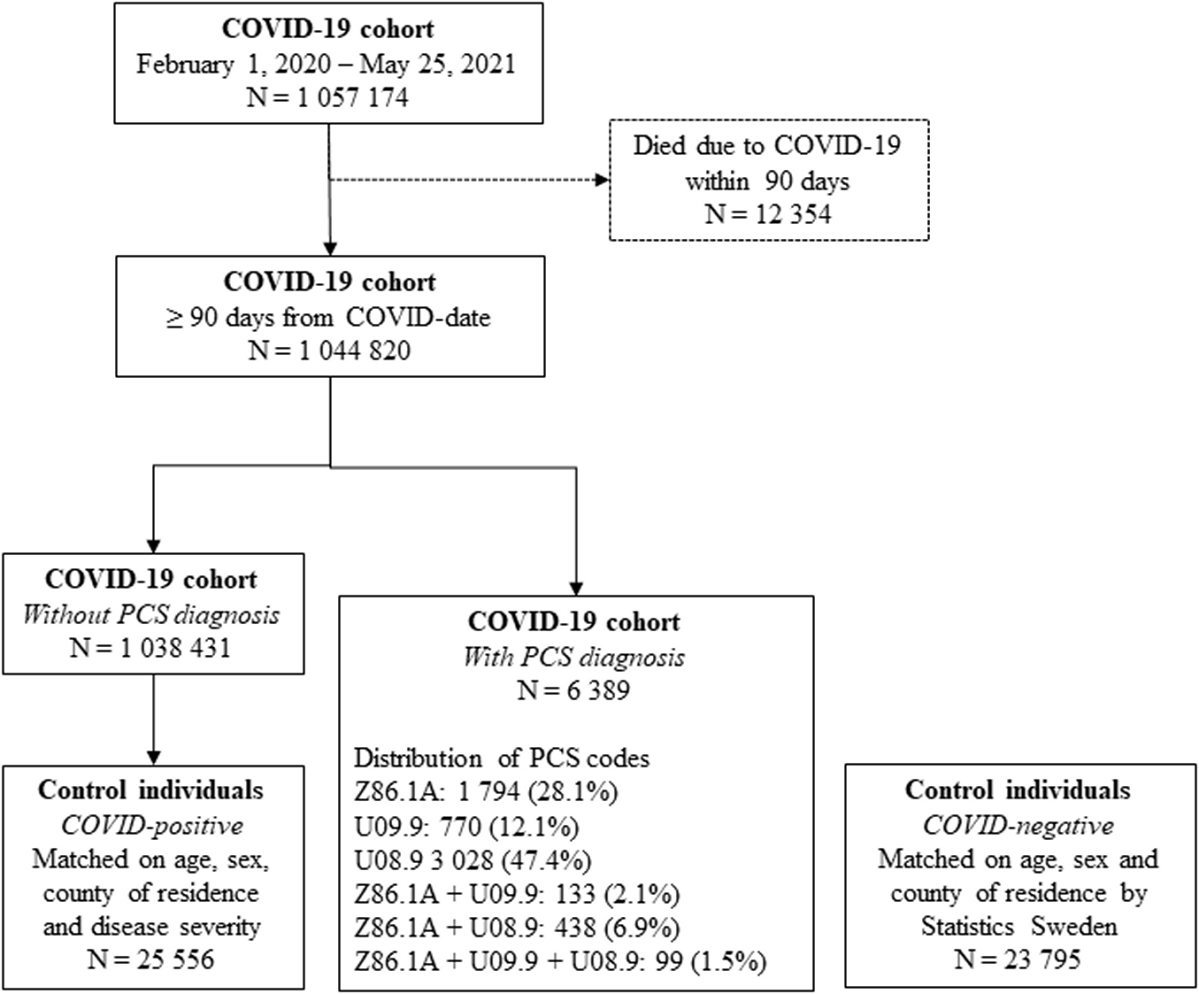
Flow chart of the COVID-19 cohort and control individuals, and distribution of post-COVID syndrome (PCS) codes used.

A total of 16 151 (1.5%) received a PCS diagnosis (online supplemental file 1). To understand the symptomatology how medical doctors use the novel ICD-10 codes for PCS, we examined the population who had received a diagnosis (first or recurrent) at least 3 months after the COVID-19 date. There were 6389 (0.61%) individuals with a healthcare visit where a PCS diagnostic code was registered from 1 044 820 COVID-19 patients 3 months post-COVID-19 (figure 1 and table 1). A majority of patients who were diagnosed with PCS had been hospitalised, or had needed intensive care, or non-invasive and mechanical ventilation (61%), compared with 4.4% in the nationwide COVID-19 cohort (table 1). However, 33% of individuals with PCS diagnosis had not had contact with outpatient or inpatient clinics during the acute phase of initial COVID-19. Internal medicine (24.7%) and infection medicine (14.2%) outpatient and inpatient clinics registered the majority of PCS diagnostic codes in Sweden (online supplemental file 1). Geographically, 39.9% of all PCS visits were administered at Stockholm county council (online supplemental file 1), a county that accounts for 23% of the Swedish population (Statistics Sweden, 2021). The distribution of comorbidities according to the Charlson comorbidity grouping for the COVID-19 cohort, PCS individuals and matched control individuals is shown in online supplemental file 1.

**Table 1:** Clinical and sociodemographic characteristics of the COVID-19 cohort and matched control individuals

Variable	COVID-19 cohort, N=1 044 820		Matched control individuals	
	No PCS, n (%)N=1 038 431	PCS, n (%)N=6389	COVID-19 negative, n (%)N=23 795	COVID-19 positive, n (%)N=25 556
Disease severity				
No contact with specialist healthcare	969 172 (93)	2138 (33)		8559 (33)
Contact with outpatient clinic	23 343 (2.2)	377 (5.9)		1478 (5.8)
Hospitalisation	37 128 (3.6)	2212 (35)		9646 (38)
Non-invasive ventilation and high-flow oxygen	4012 (0.4)	471 (7.4)		2259 (8.8)
Intensive care	1339 (0.1)	104 (1.6)		402 (1.6)
Mechanical ventilation	3437 (0.3)	1087 (17)		3212 (13)
Age (years)				
0–19	165 330 (16)	139 (2.2)	527 (2.2)	495 (1.9)
20–39	365 014 (35)	1214 (19)	4370 (18)	4705 (18)
40–59	362 067 (35)	1949 (31)	7078 (30)	7178 (28)
60–79	120 184 (12)	2132 (33)	8153 (34)	8487 (33)
80+	25 836 (2.5)	955 (15)	3667 (15)	4691 (18)
Sex				
Female	530 955 (51)	3308 (52)	12 250 (51)	14 114 (55)
Male	507 476 (49)	3081 (48)	11 545 (49)	11 442 (45)
wCCI				
0	774 599 (75)	2852 (45)	14 565 (61)	12 904 (50)
1–2	165 047 (16)	1624 (25)	5500 (23)	6304 (25)
3–4	23 187 (2.2)	731 (11)	1584 (6.7)	2639 (10)
≥5	75 598 (7.3)	1182 (19)	2146 (9.0)	3709 (15)
Education				
Tertiary	291 735 (28)	1849 (29)	7409 (31)	6960 (27)
Secondary	456 015 (44)	2868 (45)	10 993 (46)	11 359 (44)
Primary	154 443 (15)	1407 (22)	4438 (19)	6109 (24)
Missing	136 238 (13)	265 (4.1)	955 (4.0)	1128 (4.4)
Income (quintiles)				
Highest	191 058 (18)	1141 (18)	5115 (21)	4344 (17)
High	194 282 (19)	1013 (16)	4128 (17)	3845 (15)
Middle	193 135 (19)	1141 (18)	4033 (17)	4290 (17)
Low	183 159 (18)	1537 (24)	5142 (22)	6256 (24)
Lowest	150 184 (14)	1412 (22)	4720 (20)	6310 (25)
Missing	126 613 (12)	145 (2.3)	657 (2.8)	511 (2.0)

PCS, post-COVID syndromewCCIweighted Charlson Comorbidity Index

### Factors associating with receiving a PCS diagnosis by physicians in the COVID-19 cohort

**Figure 2:**
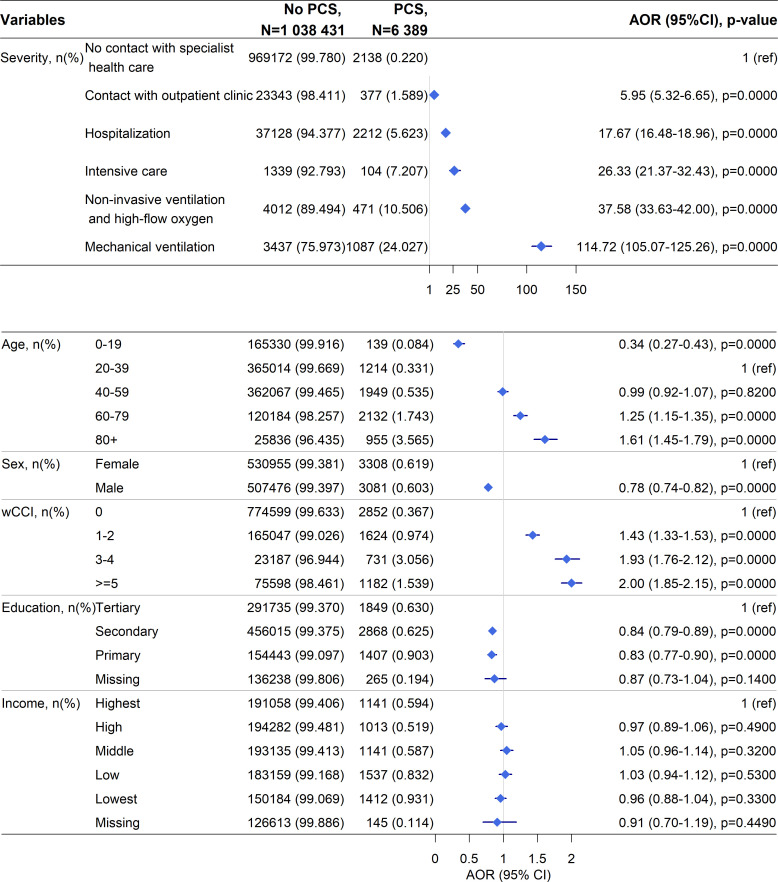
Multivariable logistic regression of association between potential risk factors (COVID-19 severity, age, sex, comorbidities, education, income) during the acute phase and receiving a PCS diagnosis during visits to outpatient and inpatient clinics at least 3 months after COVID-19 onset. All COVID-19-positive individuals are included in this analysis. The multivariable logistic regression includes all variables in the figure (ie, model 5 from online supplemental file 1). AOR, adjusted OR, PCS, post-COVID syndrome; wCCI weighted Charlson Comorbidity Index.

We estimated risk factors for PCS diagnosis first in the full cohort of COVID-19-positive individuals prior to matching for disease severity or other demographic factors. The strongest risk factor for PCS diagnosis was needing mechanical ventilation during the acute phase of COVID-19 (adjusted OR (model 5 (adjusted for age, sex, education and comorbidities) 114.7, 95% (CI) 105.1 to 125.3) (figure 2 and online supplemental file 1). In model 5 including all potential risk factors, there was an approximately 25% and 61% higher risk of being diagnosed with PCS for the age groups 60–79 and 80+ years, respectively, compared with the reference group (20–39 years). Men had a lower risk of receiving a PCS diagnosis compared with women (0.78 (0.74 to 0.82)). Having only secondary and primary education compared with tertiary education associated with a lower risk of PCS diagnosis, whereas income did not significantly associate with PCS diagnosis (figure 2). Higher wCCI associated with PCS diagnosis, and specifically having had peripheral vascular disease, cerebrovascular disease, chronic obstructive pulmonary disorder, rheumatic disease, diabetes with complications, renal disease, ascites and malignancy associated significantly with an increased risk of receiving a PCS diagnosis (online supplemental file 1).

### Clinical features prompting diagnosis with PCS by physicians in an outpatient/inpatient setting

The most frequent symptoms and signs diagnostic codes (ICD-10 R-chapter) for PCS-associated healthcare visits were dyspnoea, malaise/fatigue, abnormal findings on pulmonary diagnostic imaging and chest pain (table 2 and online supplemental file 1). When including all diagnoses, the most frequent diagnosis was hypertension; however, the majority of the individuals had this diagnosis registered before COVID-19 (online supplemental file 1).

**Table 2:** Symptomatology associated with post-COVID syndrome (PCS) diagnosis

ICD-10 codes from R-chapter (signs and symptoms)PCS cohort, N=6389	PCS visitn (% PCS individuals)	Previously diagnosedn (% PCS individuals with specific diagnosis)
Dyspnoea	856 (13.40)	166 (19.39)
Malaise and fatigue	513 (8.03)	38 (7.41)
Abnormal findings on pulmonary diagnostic imaging	272 (4.26)	12 (4.41)
Chest pain	247 (3.87)	99 (40.08)
Fever	172 (2.69)	37 (21.51)
Cough	139 (2.18)	19 (13.67)
Dysphagia	135 (2.11)	25 (18.52)
Headache	132 (2.07)	33 (25)
Abdominal pain	115 (1.80)	49 (42.61)
Dizziness	110 (1.72)	26 (23.64)
Abnormal results of pulmonary function studies	100 (1.57)	2 (2)
Pain	99 (1.55)	12 (12.12)
Palpitations	90 (1.41)	10 (11.11)
Subjective mild cognitive disorder	89 (1.39)	1 (1.12)
Skin paraesthesia	81 (1.27)	8 (9.88)
Tachycardia	76 (1.19)	2 (2.63)

ICD-10International Classification of Diseases 10th version

Diagnoses most likely associated with PCS symptomatology as seen by the clinicians, and with fewer than 10% having these diagnoses previously, were subjective mild cognitive disorder, post-viral fatigue syndrome, abnormal results of pulmonary function studies and diagnostic imaging, tachycardia, sleep disorder, malaise and fatigue, pulmonary embolism and skin paraesthesia (online supplemental file 1).

### Matched cohort study comparing PCS patients with matched COVID-19-negative and COVID-19-positive control individuals

To identify clinical features of the PCS cohort, we extended the analysis to include all visits to hospital outpatient and inpatient clinics regardless of cause, instead of only visits due to PCS, at least 3 months after COVID-19 index date (online supplemental file 1). A matched cohort study was performed comparing these individuals without COVID-19 and those with COVID-19.

We then compared the overall healthcare usage between PCS and control cohorts. The most frequent diagnosis for PCS individuals compared with matched control individuals was hypertension (26.6% vs 3.1% in matched COVID-19-negative controls and 4.8% in matched COVID-19-positive controls) (table 3 and online supplemental file 1). However, 73.5% of the PCS individuals had received this diagnosis prior to COVID-19, indicating hypertension was not a new diagnosis following COVID-19. The second most frequent ICD-10 code was dyspnoea (17.2% in PCS vs 0.4% in matched COVID-19-negative controls and 1.1% in matched COVID-19-positive controls). Dyspnoea was a new diagnosis for 76% of the PCS individuals who received this diagnosis after COVID-19.

**Table 3:** Matched cohort study identifying diagnoses associated with the post-COVID syndrome (PCS) patients compared with matched COVID-19-negative and disease severity-matched COVID-19-positive control individuals

ICD-10 codes	COVID-19-positive (PCS),N=6389		COVID-19-negative matched controls, N=23 795			COVID-19-positive matched controls, N=25 556		
	Healthcare visitn (% of PCS cohort)	Previously diagnosedn (% with diagnosis)	Healthcare visit, n (% of COVID-19-negative cohort)	Previously diagnosedn (% with diagnosis)	OR (95% CI)	Healthcare visit, n (% of COVID-19-positive cohort)	Previously diagnosedn (% with diagnosis)	OR (95% CI)
Hypertension	1700 (26.61)	1249 (73.47)	728 (3.06)	556 (76.37)	17.16 (15.23 to 19.34)	1221 (4.78)	1010 (82.72)	9.25 (8.41 to 10.16)
Dyspnoea	1099 (17.20)	261 (23.75)	89 (0.37)	32 (35.96)	54.16 (42.86 to 68.45)	286 (1.12)	99 (34.62)	18.7 (16.21 to 21.57)
Diabetes mellitus type 2	733 (11.47)	546 (74.49)	246 (1.03)	189 (76.83)	13.49 (11.52 to 15.8)	650 (2.54)	514 (79.08)	5.2 (4.64 to 5.83)
Atrial fibrillation and flutter	668 (10.46)	465 (69.61)	292 (1.23)	220 (75.34)	12.26 (10.42 to 14.41)	502 (1.96)	395 (78.69)	6.52 (5.73 to 7.41)
Heart failure	668 (10.46)	418 (62.57)	230 (0.97)	162 (70.43)	16.94 (14.1 to 20.35)	497 (1.94)	334 (67.2)	7.13 (6.24 to 8.15)
Malaise and fatigue	636 (9.95)	59 (9.28)	59 (0.25)	11 (18.64)	45.83 (34.45 to 60.98)	149 (0.58)	31 (20.81)	19.63 (16.22 to 23.75)
Chest pain	437 (6.84)	207 (47.37)	95 (0.40)	42 (44.21)	19.44 (15.31 to 24.69)	288 (1.13)	148 (51.39)	6.56 (5.62 to 7.66)
Chronic obstructive pulmonary disorder	391 (6.12)	298 (76.21)	105 (0.44)	86 (81.9)	16.45 (13.06 to 20.72)	281 (1.10)	227 (80.78)	6.23 (5.3 to 7.33)
Fever	384 (6.01)	90 (23.44)	54 (0.23)	4 (7.41)	28.44 (21.23 to 38.1)	152 (0.59)	46 (30.26)	11.1 (9.12 to 13.51)
Asthma	373 (5.84)	229 (61.39)	58 (0.24)	41 (70.69)	25.64 (19.27 to 34.12)	188 (0.74)	144 (76.6)	8.48 (7.07 to 10.16)
Old myocardial infarction	353 (5.53)	294 (83.29)	129 (0.54)	111 (86.05)	12.48 (10.02 to 15.55)	248 (0.97)	200 (80.65)	6.32 (5.33 to 7.5)
Abdominal pain	330 (5.17)	158 (47.88)	93 (0.39)	31 (33.33)	13.74 (10.86 to 17.37)	404 (1.58)	186 (46.04)	3.47 (2.98 to 4.03)
Spontaneous vertex delivery	317 (4.96)	159 (50.16)	69 (0.29)	32 (46.38)	27.7 (19.84 to 38.67)	95 (0.37)	48 (50.53)	20.07 (15.19 to 26.52)
Urinary tract infection	311 (4.87)	157 (50.48)	102 (0.43)	44 (43.14)	12.86 (10.16 to 16.27)	239 (0.94)	124 (51.88)	5.85 (4.89 to 6.99)
Abnormal findings on diagnostic imaging of lung	309 (4.84)	16 (5.18)	19 (0.08)	6 (31.58)	63.52 (39.48 to 102.2)	49 (0.19)	12 (24.49)	25.7 (18.96 to 34.83)
Pulmonary embolism	299 (4.68)	20 (6.69)	27 (0.11)	6 (22.22)	46.09 (30.4 to 69.88)	88 (0.34)	10 (11.36)	14.72 (11.5 to 18.85)
Obesity	280 (4.38)	128 (45.71)	54 (0.23)	29 (53.7)	20.8 (15.35 to 28.19)	139 (0.54)	79 (56.83)	8.95 (7.22 to 11.08)
Hypothyroidism	248 (3.88)	172 (69.35)	87 (0.37)	64 (73.56)	11.54 (8.97 to 14.86)	141 (0.55)	102 (72.34)	7.59 (6.12 to 9.4)
Anaemia	242 (3.79)	79 (32.64)	82 (0.34)	24 (29.27)	11.89 (9.18 to 15.4)	186 (0.73)	69 (37.1)	5.49 (4.51 to 6.69)
Hyperlipidaemia, unspecified	229 (3.58)	109 (47.6)	114 (0.48)	60 (52.63)	7.83 (6.23 to 9.85)	172 (0.67)	103 (59.88)	5.65 (4.61 to 6.92)

ICD-10International Classification of Diseases 10th version

The diagnoses that were present at a significantly higher proportion in the PCS cohort compared with the COVID-19-negative/positive matched control individuals in the matched cohort study were listed according to percentage of PCS individuals who had not received this diagnosis prior to SARS-CoV-2 infection (online supplemental file 1).

## Discussion

Currently, the pathophysiology and understanding of mechanisms of PCS are evolving including persistent infection, autoimmunity or dysfunction of blood clotting.^19 20 24^ The risk factors and clinical diagnostic features in COVID-19 patients with long-term sequelae that result in a PCS diagnosis have remained elusive, and most of the literature on PCS is based on follow-up studies of hospitalised patients^25 26^ and questionnaire data.^7 10 12 27 28^ A recent study was published using healthcare data covering 25% of the Israeli population^29^; however, no studies have been performed on a total country’s population including all individuals who have a laboratory-verified positive test for SARS-CoV-2 in the country regardless of initial COVID-19 severity. This approach allows us to attribute diagnosis of PCS and related symptomatology, and describe the characteristics of physicians who use the PCS diagnosis with regard to clinical specialty and geography.

In the total population of Sweden, we examined all laboratory-verified test-positive SARS-CoV-2 individuals and discovered that the initial COVID-19 severity was the strongest associating factor for physicians assigning a PCS diagnosis in an outpatient/inpatient setting. We found that Swedish physicians during the first and second waves of the pandemic primarily associated dyspnoea and fatigue among other symptoms with PCS. In addition, we identified several diagnoses that were present prior to COVID-19, and thereby not likely associated with clinical features of PCS.

It is noteworthy that the PCS code is novel and likely used differently across practices and geographical location. This is also supported by our results and in our study, we see the highest usage in the Stockholm metropolitan area. Furthermore, there likely is not yet a harmonised national standard across different locations on how to use ICD-10 codes for PCS. Our study provides insight into how these novel codes are used in Sweden and the symptomatology that prompts healthcare providers and medical doctors to assign the code. Yet, the novel ICD-10 codes likely do not capture diagnoses for all PCS patients.

The main strength of our study is the use of the entire population of laboratory-verified SARS-CoV-2 test-positive individuals in Sweden (approximately 1 million) during 1 February 2020–25 May 2021 with historical data from visits to outpatient/inpatient clinics from 1997. This approach allows population-based estimates and offers a clinical perspective, which is complementary to self-reported symptoms in questionnaire-based studies, with the added benefit of identifying the specific diagnoses that were new following COVID-19 onset. The Nordic countries are unique in having PINs for every individual in the population, with Sweden having a numerically larger population, providing the infrastructure that is needed for our study.^30^

A weakness is the lack of primary care data, which are not registered centrally in Sweden; and additionally, the access to outpatient/inpatient clinics and follow-up was not standardised. The PCS incidence is most likely conceivably larger than what is represented in the data, due to initial physician lack of awareness of PCS and associated novel ICD-10 codes for diagnosis. Therefore, we examined how clinicians attribute the code to patients who received the PCS diagnosis. This is made obvious in our study where the proportion of individuals diagnosed with PCS by a physician in an outpatient/inpatient setting during the early pandemic phases was 0.61%. This is considerably lower than reported in other studies ranging from 14.1%,^7^ 12.5%,^28^ 6.2%^15^ to 3.3% of the UK population according to the UK Office for National Statistics.^31^

The initial COVID-19 severity was a strong associating factor for physicians assigning a PCS diagnosis to patients in our study. This is corroborated in earlier reports.^6^,^8^ Another associating factor for physicians assigning a PCS diagnosis in our study was female sex, as found in other studies.^15 25 26 31 32^

Dyspnoea, malaise/fatigue/post-viral fatigue syndrome and abnormal pulmonary imaging/findings were some of the most prominent clinical features that physicians associated with PCS. These clinical features were also enhanced in the COVID-19 patients with PCS, even when compared with COVID-19 patients without a PCS diagnosis but with the same initial severity of disease. The majority of the PCS patients had not received these diagnoses prior to COVID-19 onset. Since we compare intensive care unit (ICU) patients with PCS with ICU patients without PCS, our findings cannot be attributed to PICS only.^16^ The PCS cohort has a characteristic profile compared with the remaining COVID-19 cohort. Although most of the literature on PCS is based on follow-up studies of hospitalised patients,^25 26^ our findings are in line with the diverse symptomatology previously reported by questionnaire data,^7 12 27 28^ and recently also with electronic health record data from Veteran’s affairs data.^6 9^ Furthermore, systematic reviews and meta-analyses identify fatigue and dyspnoea as common long-term symptoms.^12^,^14^

In our study, pulmonary embolism was present in 5% of the PCS cohort, which was a new diagnosis to the majority; and the OR was 15-fold higher when compared with disease severity-matched COVID-19-positive control individuals. In a previous study, we found that the risk of pulmonary embolism was highest for COVID-19 patients in need of intensive care, and still significantly increased in the time period of 3–6 months after COVID-19 indicating a potential association between pulmonary embolism and dyspnoea for this particular patient group.^19^ This indicates that the PCS cohort has a distinct symptomatic/disease profile as seen by the clinicians from the remaining COVID-19 cohort.

While the mechanisms of PCS are emerging, there are several potential likely disease mechanisms.^24^ For example, COVID-19 has been demonstrated to damage vasculature.^33^ Consequently, defects in blood supply and tissue oxygenation may contribute to more severe clinical picture particularly in patients where pulmonary damage through severe disease has occurred, and in patients who have dysfunction of peripheral vasculature. Furthermore, independent of disease severity, the dysfunction of the autonomic nervous system may be caused by autoimmunity, epitope spreading or direct persistent infection.^34 35^ Interestingly, in our study, 2% of individuals had SARS-CoV-2 detected in the same visit where they received a PCS diagnosis by a physician, raising the possibility of reinfection or persistent infection. Similarly, the clinical picture in PCS may relate to novel infections or opportunistic secondary infections such as those caused by *Echerichia coli* or other bacterial infections as we observe are enhanced in the PCS cohort in our study, or reactivation of latent pathogens including Epstein-Barr or varicella virus.^24 34 35^

In summary, we found that Swedish physicians associated the following with PCS: respiratory dysfunction, neurological and cognitive disorders, and cardiac and circulatory dysfunction. Initial COVID-19 severity was the strongest associating factor for receiving a PCS diagnosis by a physician. More studies are required to further characterise this emerging syndrome, and particularly in a primary care setting.

## supplementary material

10.1136/bmjph-2023-000336 online supplemental file 1

## Data Availability

Data may be obtained from a third party and are not publicly available.
