# Supplementary material for: How do clinicians use post-COVID syndrome diagnosis? Analysis of clinical features in a Swedish COVID-19 cohort with 18 months’ follow-up: a national observational cohort and matched cohort study
Source: BMJ Public Health. 2024 Mar 25;2(1):e000336. doi: 10.1136/bmjph-2023-000336 (PMC11816610; doi:10.1136/bmjph-2023-000336)
Supplement: online supplemental file 1 [file bmjph-2-1-s001.pdf]

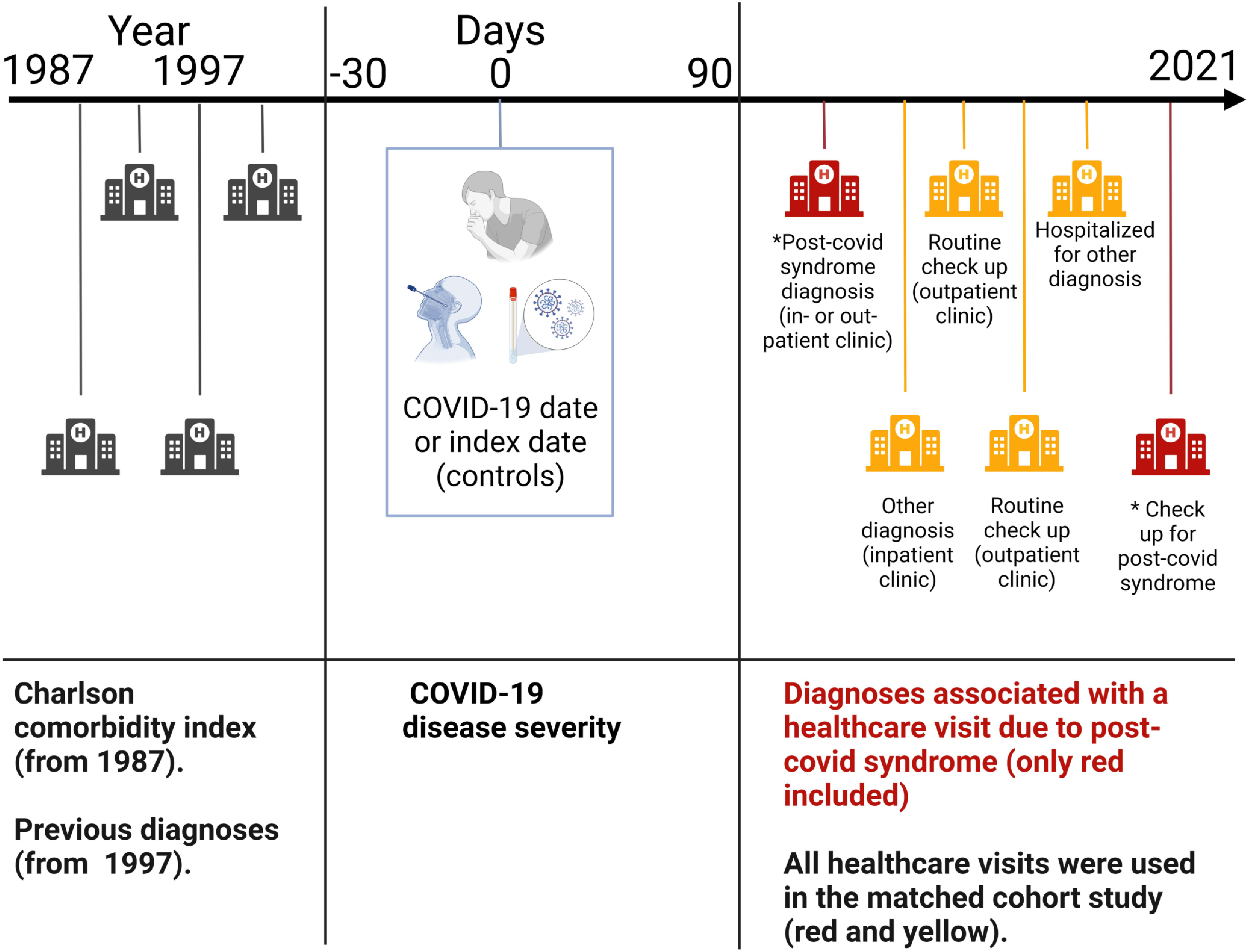

Supplementary Figure 1. Description of the data and which outpatient and inpatient healthcare visits were included in the different parts of the analyses. Created with BioRender.

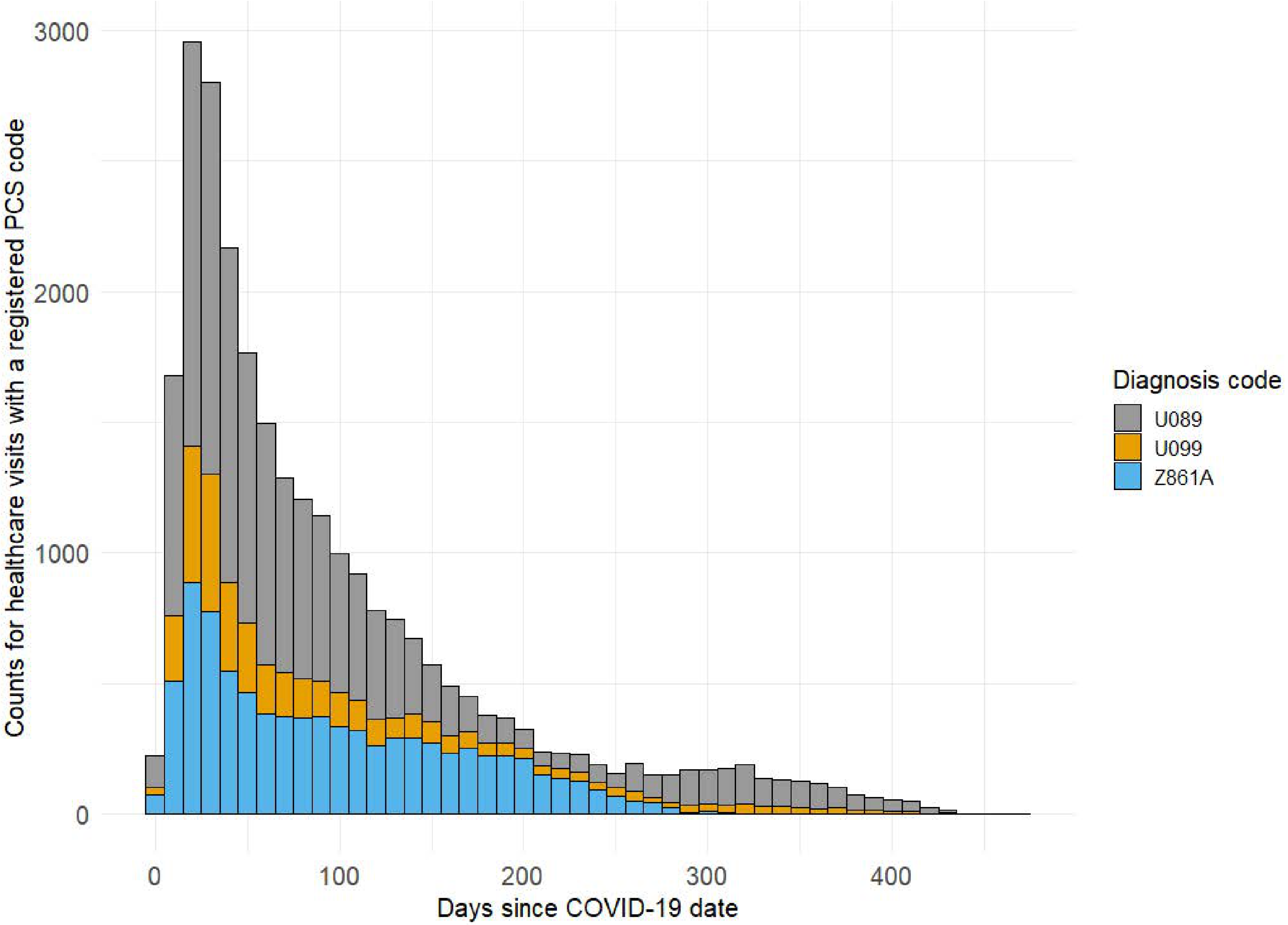

Supplementary Figure 2. Description of healthcare visits where any of the three PC codes were registered, regardless of whether it was a first or a recurrent diagnosis.

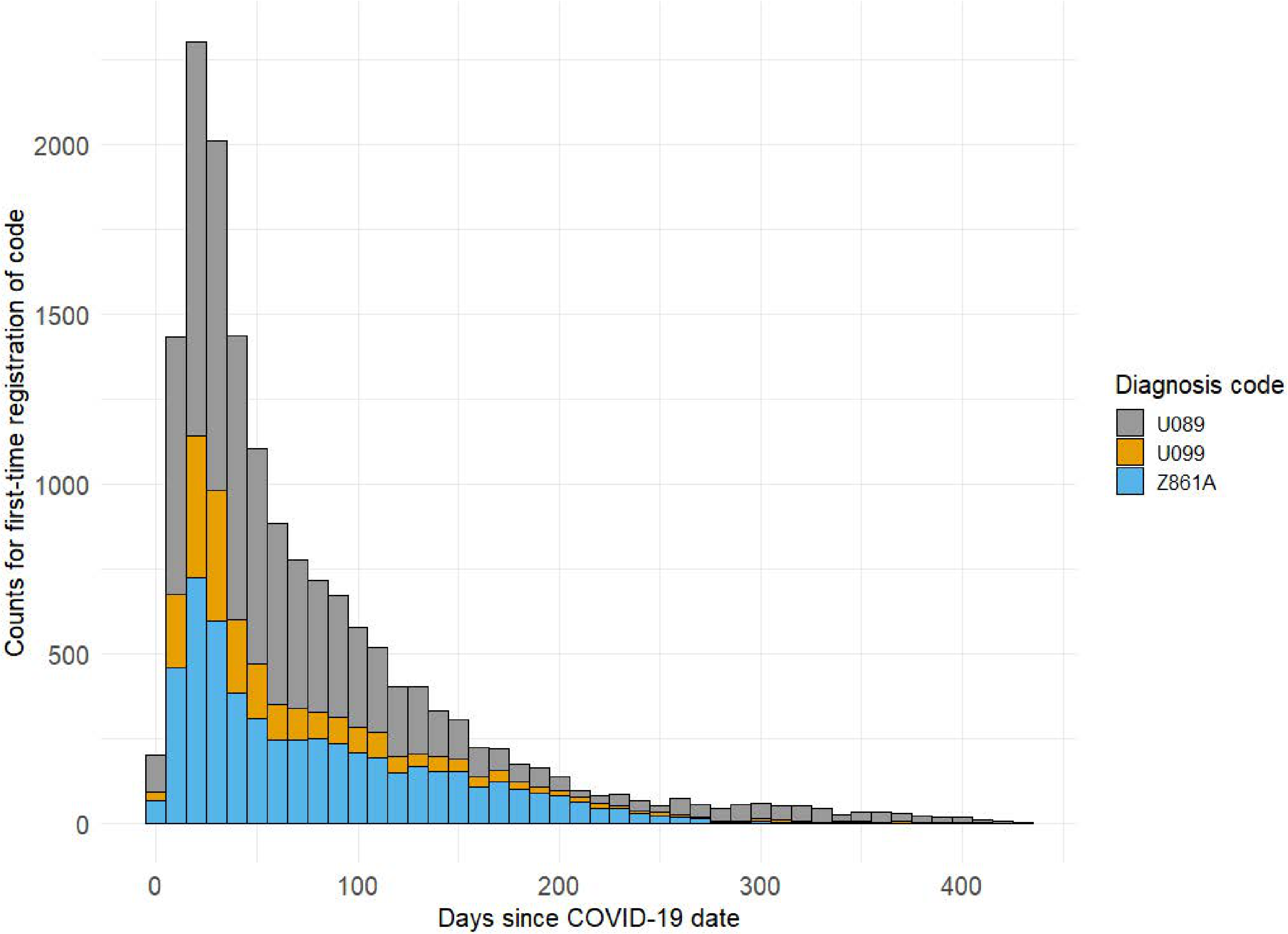

Supplementary Figure 3. Description of healthcare visits including the first-time registration of one of the three PCS codes. Recurrent visits where the same code was registered, are not shown here.

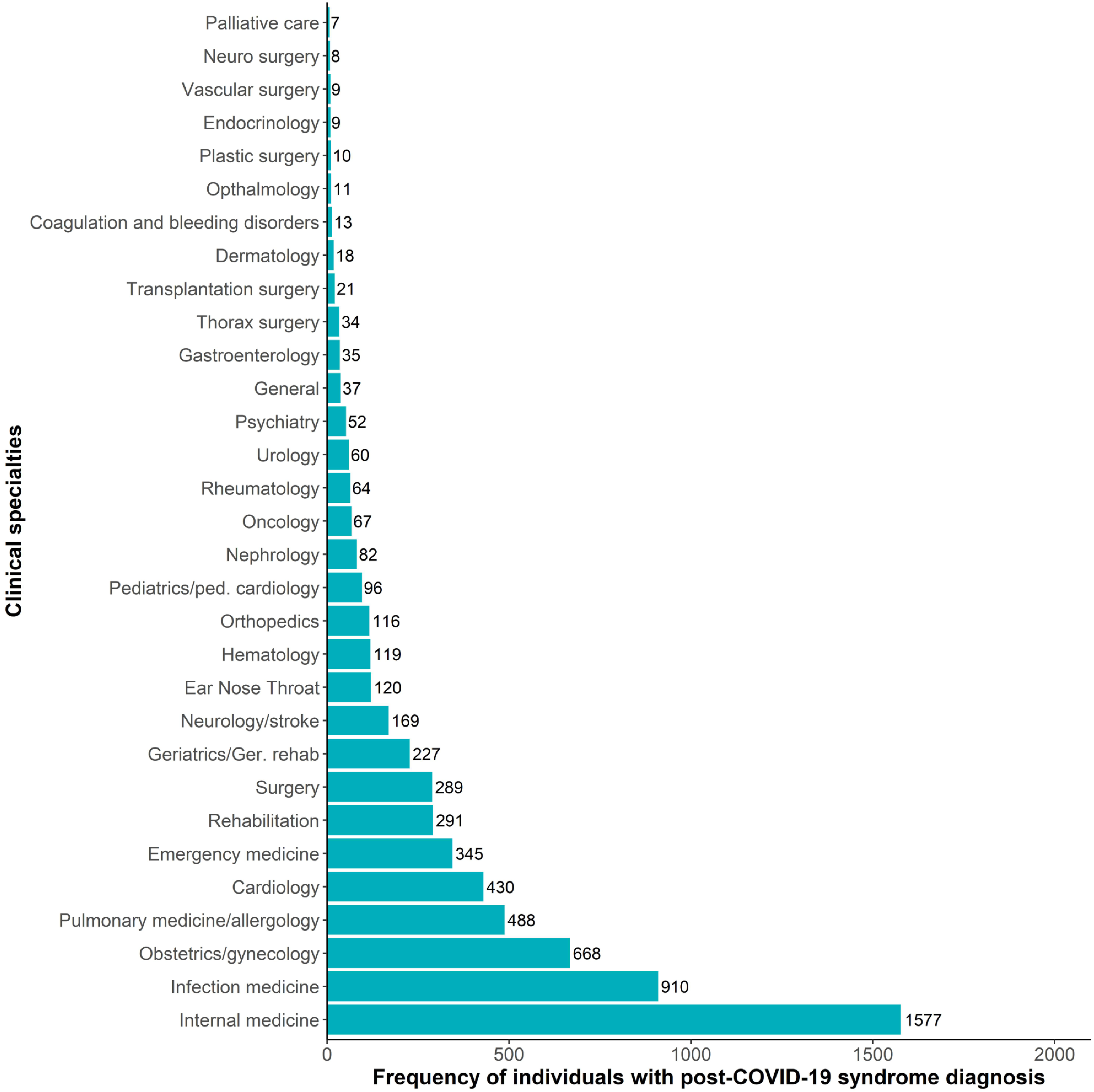

Supplementary Figure 4. Description of which Swedish outpatient and inpatient clinical specialties assign a PCS diagnosis code.

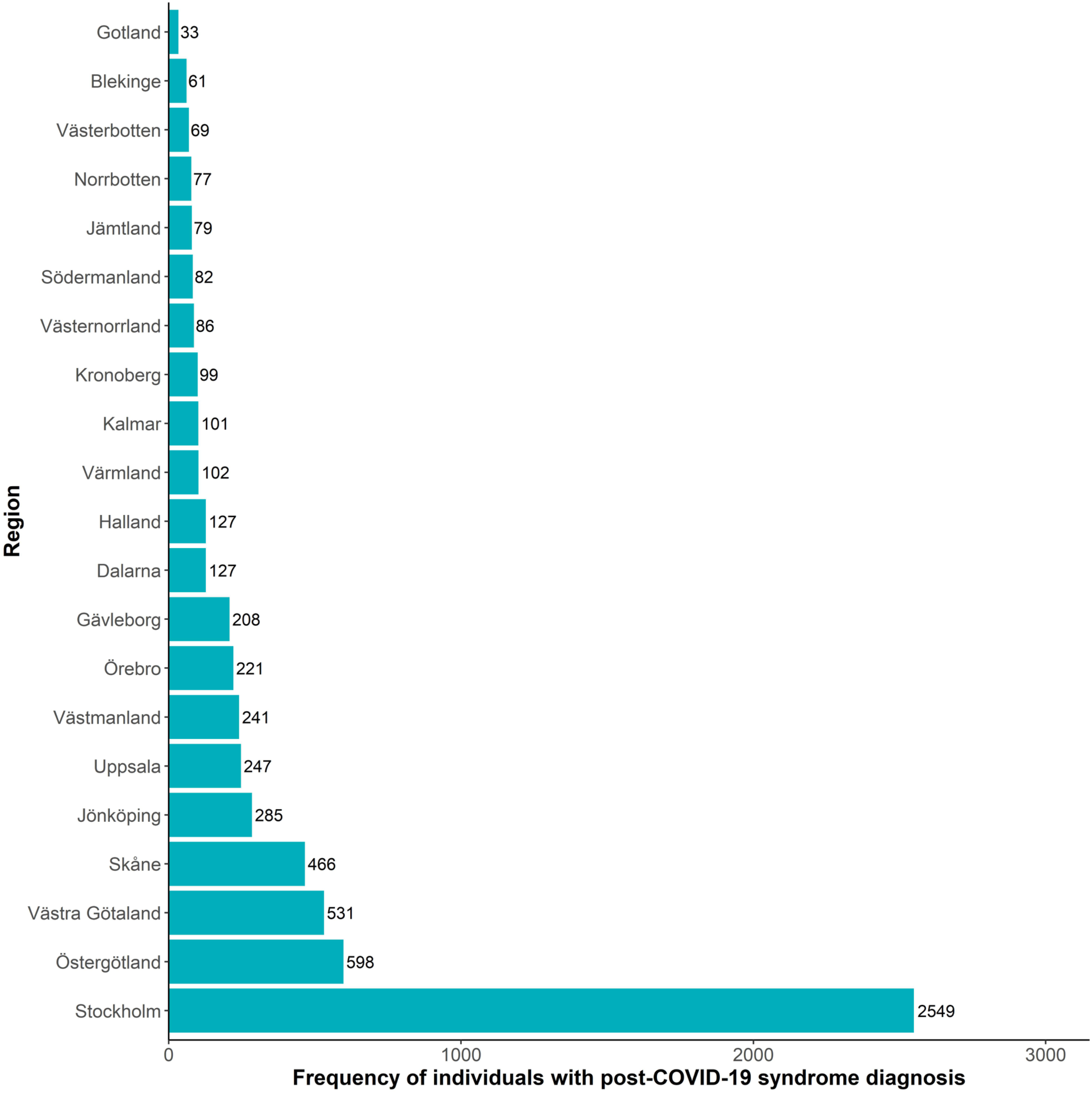

Supplementary Figure 5. Description of which county councils (regions) in Sweden with outpatient or inpatient clinics that assign a PCS diagnosis code.

Supplementary Table 1. The historical diagnosis codes that were searched for in historical outpatient and inpatient registry data. This was for the PCS patients and matched control individuals (COVID-negative and COVID-positive)

ICD10 code	Diagnosis codes to look for in historical registry data
B962	B962
D509	D50*, D51*, D52*, D53*, D649
D649	D649
E039	E03*
E118	E11*
E119	E11*
E669	E65*, E66*, E67*, E68*
E785	E78*
F039	F00*, F01*, F02*, F03*, F067
F067	F00*, F01*, F02*, F03*, F067
F419	F41*
G409	G40*, G41*
G473	G473
G479	G479
G819	G80*, G81*, G82*, G83*
G933	G933
I109	I10*, I11*, I12*, I13*, I15*
I209	I20*
I252	I21*, I22*, I23*, I24*, I25*
I259	I21*, I22*, I23*, I24*, I25*
I269	I26*
I350	I350
I480	I48*
I482	I48*
I489	I48*
I509	I50*
I693	I63*, I693, I698, I694,
J159	J13*, J14*, J15*, J170, J18*
J189	J12*, J13*, J14*, J15*, J16*, J17*, J18*
J441	J44*
J449	J44*
J459	J45*, J46*
J841	J841
J909	J90*, J91*
J969	J96*
N109	N10*, N11*, N12*
N179	N17*
N183	N17*, N18*

N184	N17*, N18*
N185	N17*, N18*
N189	N17*, N18*
N390	N110, N111, N118, N119, N129, N209, N300, N309, N390
N409	N40*
O701	O70*
O800A	O800*
R000	R000
R002	R002
R059	R059
R060	R060
R074	R074
R104X	R104X
R139	R139
R202	R202
R418A	R418A
R429	R429
R509	R509
R519	R519
R529	R529
R539	R539
R919	R919
R942	R942

Supplementary Table 2. The distribution of Charlson comorbidity groups in the COVID-19 positive cohort without or with post-covid syndrome (PCS) and the COVID-negative control individuals. The Charlson comorbidity groups were determined up to 1 month prior to the COVID-19 or index date. PCS post-covid syndrome.

**Charlson comorbidity groups**	**COVID-19 cohort (N=1,044,820)**		**Matched control individuals**	
	**No PCS, n (%), N = 1,038,431**	**PCS, n (%), N = 6,389**	**COVID-positive, n (%), N = 25,556**	**COVID-negative, n (%), N = 23,795**
Myocardial infarction				
No	1,024,244 (98.63%)	5,846 (91.50%)	23,779 (93.05%)	22,711 (95.44%)
Yes	14,187 (1.37%)	543 (8.50%)	1,777 (6.95%)	1,084 (4.56%)
Congestive heart failure				
No	1,025,290 (98.73%)	5,696 (89.15%)	23,451 (91.76%)	22,741 (95.57%)
Yes	13,141 (1.27%)	693 (10.85%)	2,105 (8.24%)	1,054 (4.43%)
Peripheral vascular disease				
No	1,031,325 (99.32%)	6,093 (95.37%)	24,579 (96.18%)	23,260 (97.75%)
Yes	7,106 (0.68%)	296 (4.63%)	977 (3.82%)	535 (2.25%)
Cerebrovascular disease				
No	1,015,957 (97.84%)	5,769 (90.30%)	22,980 (89.92%)	22,229 (93.42%)
Yes	22,474 (2.16%)	620 (9.70%)	2,576 (10.08%)	1,566 (6.58%)
Chronic obstructive pulmonary disease				
No	1,029,942 (99.18%)	5,935 (92.89%)	24,246 (94.87%)	23,186 (97.44%)
Yes	8,489 (0.82%)	454 (7.11%)	1,310 (5.13%)	609 (2.56%)
Chronic other pulmonary disease				
No	961,200 (92.56%)	5,570 (87.18%)	23,232 (90.91%)	22,530 (94.68%)
Yes	77,231 (7.44%)	819 (12.82%)	2,324 (9.09%)	1,265 (5.32%)

Charlson comorbidity groups	COVID-19 cohort (N=1,044,820)		Matched control individuals	
	**No PCS, n (%), N = 1,038,431**	**PCS, n (%), N = 6,389**	**COVID-positive, n (%), N = 25,556**	**COVID-negative, n (%), N = 23,795**
Rheumatic disease				
No	1,009,276 (97.19%)	5,836 (91.34%)	23,882 (93.45%)	22,705 (95.42%)
Yes	29,155 (2.81%)	553 (8.66%)	1,674 (6.55%)	1,090 (4.58%)
Dementia				
No	1,030,964 (99.28%)	6,222 (97.39%)	24,538 (96.02%)	23,409 (98.38%)
Yes	7,467 (0.72%)	167 (2.61%)	1,018 (3.98%)	386 (1.62%)
Hemiplegia				
No	1,032,682 (99.45%)	6,232 (97.54%)	24,964 (97.68%)	23,561 (99.02%)
Yes	5,749 (0.55%)	157 (2.46%)	592 (2.32%)	234 (0.98%)
Diabetes without chronic complication				
No	1,037,384 (99.90%)	6,367 (99.66%)	25,491 (99.75%)	23,765 (99.87%)
Yes	1,047 (0.10%)	22 (0.34%)	65 (0.25%)	30 (0.13%)
Diabetes with chronic complication				
No	1,026,602 (98.86%)	5,952 (93.16%)	24,176 (94.60%)	23,212 (97.55%)
Yes	11,829 (1.14%)	437 (6.84%)	1,380 (5.40%)	583 (2.45%)
Renal disease				
No	1,029,165 (99.11%)	5,922 (92.69%)	24,326 (95.19%)	23,280 (97.84%)
Yes	9,266 (0.89%)	467 (7.31%)	1,230 (4.81%)	515 (2.16%)
Mild liver disease				
No	1,030,789 (99.26%)	6,270 (98.14%)	25,197 (98.60%)	23,572 (99.06%)
Yes	7,642 (0.74%)	119 (1.86%)	359 (1.40%)	223 (0.94%)

Charlson comorbidity groups	COVID-19 cohort (N=1,044,820)		Matched control individuals	
	No PCS, n (%), N = 1,038,431	PCS, n (%), N = 6,389	COVID-positive, n (%), N = 25,556	COVID-negative, n (%), N = 23,795
Ascites				
No	1,037,888 (99.95%)	6,370 (99.70%)	25,523 (99.87%)	23,769 (99.89%)
Yes	543 (0.05%)	19 (0.30%)	33 (0.13%)	26 (0.11%)
Severe liver disease				
No	1,037,628 (99.92%)	6,357 (99.50%)	25,459 (99.62%)	23,759 (99.85%)
Yes	803 (0.08%)	32 (0.50%)	97 (0.38%)	36 (0.15%)
Peptic ulcer disease				
No	1,029,007 (99.09%)	6,147 (96.21%)	24,715 (96.71%)	23,348 (98.12%)
Yes	9,424 (0.91%)	242 (3.79%)	841 (3.29%)	447 (1.88%)
Malignancy				
No	972,514 (93.65%)	5,250 (82.17%)	21,333 (83.48%)	20,117 (84.54%)
Yes	65,917 (6.35%)	1,139 (17.83%)	4,223 (16.52%)	3,678 (15.46%)
Metastatic solid tumor				
No	1,032,631 (99.44%)	6,221 (97.37%)	25,029 (97.94%)	23,479 (98.67%)
Yes	5,800 (0.56%)	168 (2.63%)	527 (2.06%)	316 (1.33%)
Aids				
No	975,108 (93.90%)	5,828 (91.22%)	23,689 (92.69%)	22,426 (94.25%)
Yes	63,323 (6.10%)	561 (8.78%)	1,867 (7.31%)	1,369 (5.75%)

Supplementary Table 3. Variables associated with receiving a PCS diagnosis code. All COVID-positive individuals (COVID-19 cohort) are included in this analysis and a step-wise univariable (model 0) and multivariable logistic regression models used to determine which variables associate with receiving a PCS diagnosis code at any visit to outpatient or inpatient clinics at least 3 months after COVID-19 date. The Charlson comorbidity groups were determined up to 1 month prior to the COVID-19 or index date.

Post-COVID-19 Syndrome	Levels	No, n (%)	Yes, n (%)	Model 0, OR (95%CI)	Model 1, OR (95%CI)	Model 2, OR (95%CI)	Model 3, OR (95%CI)	Model 4, OR (95%CI)	Model 5, OR (95%CI)
**Severity**	No contact with specialist health care	969172 (99.8)	2138 (0.2)	-	-	-	-	-	-
	Contact with outpatient clinic	23343 (98.4)	377 (1.6)	7.32 (6.56-8.17, p<0.001)	6.23 (5.58-6.96, p<0.001)	5.92 (5.30-6.62, p<0.001)	5.94 (5.31-6.64, p<0.001)	5.95 (5.32-6.65, p<0.001)	5.95 (5.32-6.65, p<0.001)
	Hospitalization	37128 (94.4)	2212 (5.6)	27.01 (25.43-28.69, p<0.001)	19.08 (17.81-20.43, p<0.001)	17.40 (16.23-18.65, p<0.001)	17.63 (16.44-18.90, p<0.001)	17.57 (16.38-18.84, p<0.001)	17.67 (16.48-18.96, p<0.001)
	Intensive care	1339 (92.8)	104 (7.2)	35.21 (28.71-43.17, p<0.001)	27.79 (22.59-34.18, p<0.001)	25.75 (20.92-31.71, p<0.001)	26.26 (21.33-32.34, p<0.001)	26.03 (21.14-32.05, p<0.001)	26.33 (21.37-32.43, p<0.001)
	Non-invasive ventilation and high-flow oxygen	4012 (89.5)	471 (10.5)	53.22 (47.94-59.08, p<0.001)	39.51 (35.41-44.10, p<0.001)	36.90 (33.04-41.21, p<0.001)	37.47 (33.54-41.86, p<0.001)	37.30 (33.38-41.67, p<0.001)	37.58 (33.63-42.00, p<0.001)
	Mechanical ventilation	3437 (76.0)	1087 (24.0)	143.37 (132.30-155.36, p<0.001)	117.10 (107.39-127.69, p<0.001)	111.96 (102.62-122.15, p<0.001)	114.36 (104.77-124.83, p<0.001)	113.11 (103.62-123.47, p<0.001)	114.72 (105.07-125.26, p<0.001)
**Age, years**	0-19	165330 (99.9)	139 (0.1)	0.25 (0.21-0.30, p<0.001)	0.32 (0.27-0.38, p<0.001)	0.31 (0.26-0.37, p<0.001)	0.33 (0.27-0.41, p<0.001)	0.33 (0.26-0.41, p<0.001)	0.34 (0.27-0.43, p<0.001)
	20-39	365014 (99.7)	1214 (0.3)	-	-	-	-	-	-
	40-59	362067 (99.5)	1949 (0.5)	1.62 (1.51-1.74, p<0.001)	1.03 (0.96-1.12, p=0.369)	1.00 (0.93-1.08, p=0.991)	0.99 (0.92-1.07, p=0.802)	0.99 (0.92-1.07, p=0.847)	0.99 (0.92-1.07, p=0.819)
	60-79	120184 (98.3)	2132 (1.7)	5.33 (4.97-5.72, p<0.001)	1.47 (1.36-1.59, p<0.001)	1.24 (1.14-1.34, p<0.001)	1.25 (1.15-1.35, p<0.001)	1.23 (1.13-1.34, p<0.001)	1.25 (1.15-1.35, p<0.001)

Post-COVID-19 Syndrome	Levels	No, n (%)	Yes, n (%)	Model 0, OR (95%CI)	Model 1, OR (95%CI)	Model 2, OR (95%CI)	Model 3, OR (95%CI)	Model 4, OR (95%CI)	Model 5, OR (95%CI)
	80+	25836 (96.4)	955 (3.6)	11.11 (10.20-12.11, p<0.001)	2.17 (1.97-2.39, p<0.001)	1.59 (1.44-1.76, p<0.001)	1.62 (1.46-1.80, p<0.001)	1.59 (1.43-1.76, p<0.001)	1.61 (1.45-1.79, p<0.001)
**Sex**	Female	530955 (99.4)	3308 (0.6)	-	-	-	-	-	-
	Male	507476 (99.4)	3081 (0.6)	0.97 (0.93-1.02, p=0.303)	0.76 (0.72-0.80, p<0.001)	0.77 (0.73-0.82, p<0.001)	0.78 (0.74-0.83, p<0.001)	0.77 (0.73-0.81, p<0.001)	0.78 (0.74-0.82, p<0.001)
**wCCI**	0	774599 (99.6)	2852 (0.4)	-	-	-	-	-	-
	1-2	165047 (99.0)	1624 (1.0)	2.67 (2.51-2.84, p<0.001)	-	1.42 (1.33-1.52, p<0.001)	1.43 (1.34-1.53, p<0.001)	1.42 (1.33-1.52, p<0.001)	1.43 (1.33-1.53, p<0.001)
	3-4	23187 (96.9)	731 (3.1)	8.56 (7.89-9.30, p<0.001)	-	1.92 (1.74-2.11, p<0.001)	1.93 (1.76-2.13, p<0.001)	1.92 (1.75-2.11, p<0.001)	1.93 (1.76-2.12, p<0.001)
	>=5	75598 (98.5)	1182 (1.5)	4.25 (3.97-4.55, p<0.001)	-	1.99 (1.84-2.15, p<0.001)	2.00 (1.85-2.16, p<0.001)	1.99 (1.84-2.15, p<0.001)	2.00 (1.85-2.15, p<0.001)
**Education**	Tertiary	291735 (99.4)	1849 (0.6)	-	-	-	-	-	-
	Secondary	456015 (99.4)	2868 (0.6)	0.99 (0.94-1.05, p=0.797)	-	-	0.84 (0.79-0.89, p<0.001)	-	0.84 (0.79-0.89, p<0.001)
	Primary	154443 (99.1)	1407 (0.9)	1.44 (1.34-1.54, p<0.001)	-	-	0.83 (0.77-0.89, p<0.001)	-	0.83 (0.77-0.90, p<0.001)
	Missing	136238 (99.8)	265 (0.2)	0.31 (0.27-0.35, p<0.001)	-	-	0.83 (0.71-0.97, p=0.017)	-	0.87 (0.73-1.04, p=0.138)
**Income (quintiles)**	Highest	191058 (99.4)	1141 (0.6)	-	-	-	-	-	-
	High	194282 (99.5)	1013 (0.5)	0.87 (0.80-0.95, p=0.002)	-	-	-	0.95 (0.87-1.04, p=0.282)	0.97 (0.89-1.06, p=0.490)
	Middle	193135 (99.4)	1141 (0.6)	0.99 (0.91-1.07, p=0.797)	-	-	-	1.02 (0.94-1.11, p=0.662)	1.05 (0.96-1.14, p=0.318)

**Post-COVID-19 Syndrome**	**Levels**	**No, n (%)**	**Yes, n (%)**	**Model 0, OR (95%CI)**	**Model 1, OR (95%CI)**	**Model 2, OR (95%CI)**	**Model 3, OR (95%CI)**	**Model 4, OR (95%CI)**	**Model 5, OR (95%CI)**
	Low	183159 (99.2)	1537 (0.8)	1.41 (1.30-1.52, p<0.001)	-	-	-	0.99 (0.91-1.07, p=0.767)	1.03 (0.94-1.12, p=0.532)
	Lowest	150184 (99.1)	1412 (0.9)	1.57 (1.46-1.70, p<0.001)	-	-	-	0.91 (0.84-0.99, p=0.035)	0.96 (0.88-1.04, p=0.326)
	Missing	126613 (99.9)	145 (0.1)	0.19 (0.16-0.23, p<0.001)	-	-	-	0.90 (0.72-1.13, p=0.372)	0.91 (0.70-1.19, p=0.499)

Supplementary Table 4. Variables associated with receiving a PCS diagnosis code with individual Charlson comorbidity groups included. All COVID-positive individuals are included in this analysis using model 0 (univariable) and model 5 (multivariable) logistic regression models to determine which individual Charlson comorbidity groups associate with receiving a PCS diagnosis code at any visit to outpatient or inpatient clinics at least 3 months after COVID-19 date. The Charlson comorbidity groups were determined up to 1 month prior to the COVID-19 or index date.

Dependent: Post-COVID-19 Syndrome	Levels	COVID-positive, No PCS, n (%) N= 1,038,431	COVID-positive, PCS, n (%) N= 6389	COR (95% CI, p-value)	AOR (95% CI, p-value)
Severity	No contact with specialist health care	969172 (99.8)	2138 (0.2)	-	-
	Contact with outpatient clinic	23343 (98.4)	377 (1.6)	7.32 (6.55-8.16, p<0.001)	5.93 (5.30-6.62, p<0.001)
	Hospitalization	37128 (94.4)	2212 (5.6)	27.01 (25.43-28.69, p<0.001)	17.02 (15.85-18.28, p<0.001)
	Intensive care	1339 (92.8)	104 (7.2)	35.21 (28.55-42.95, p<0.001)	25.44 (20.52-31.20, p<0.001)
	Non-invasive ventilation and high-flow oxygen	4012 (89.5)	471 (10.5)	53.22 (47.89-59.02, p<0.001)	36.53 (32.64-40.82, p<0.001)
	Mechanical ventilation	3437 (76.0)	1087 (24.0)	143.37 (132.27-155.33, p<0.001)	113.68 (104.05-124.16, p<0.001)
Age, years	0-19	165330 (99.9)	139 (0.1)	0.25 (0.21-0.30, p<0.001)	0.34 (0.27-0.43, p<0.001)
	20-39	365014 (99.7)	1214 (0.3)	-	-
	40-59	362067 (99.5)	1949 (0.5)	1.62 (1.51-1.74, p<0.001)	0.99 (0.91-1.06, p=0.731)
	60-79	120184 (98.3)	2132 (1.7)	5.33 (4.97-5.73, p<0.001)	1.19 (1.09-1.30, p<0.001)
	80+	25836 (96.4)	955 (3.6)	11.11 (10.20-12.11, p<0.001)	1.44 (1.29-1.62, p<0.001)
Sex	Female	530955 (99.4)	3308 (0.6)	-	-
	Male	507476 (99.4)	3081 (0.6)	0.97 (0.93-1.02, p=0.303)	0.77 (0.73-0.82, p<0.001)
wCCI	0	774599 (99.6)	2852 (0.4)	-	-
	1-2	165047 (99.0)	1624 (1.0)	2.67 (2.51-2.84, p<0.001)	1.24 (1.14-1.35, p<0.001)

Dependent: Post-COVID-19 Syndrome	Levels	COVID-positive, No PCS, n (%) N= 1,038,431	COVID-positive, PCS, n (%) N= 6389	COR (95% CI, p-value)	AOR (95% CI, p-value)
	3-4	23187 (96.9)	731 (3.1)	8.56 (7.88-9.29, p<0.001)	1.40 (1.22-1.61, p<0.001)
	>=5	75598 (98.5)	1182 (1.5)	4.25 (3.97-4.54, p<0.001)	1.29 (1.05-1.60, p=0.017)
Education	Tertiary	291735 (99.4)	1849 (0.6)	-	-
	Secondary	456015 (99.4)	2868 (0.6)	0.99 (0.94-1.05, p=0.797)	0.83 (0.78-0.88, p<0.001)
	Primary	154443 (99.1)	1407 (0.9)	1.44 (1.34-1.54, p<0.001)	0.82 (0.76-0.88, p<0.001)
	Missing	136238 (99.8)	265 (0.2)	0.31 (0.27-0.35, p<0.001)	0.86 (0.72-1.03, p=0.102)
Income (quintiles)	Highest	191058 (99.4)	1141 (0.6)	-	-
	High	194282 (99.5)	1013 (0.5)	0.87 (0.80-0.95, p=0.002)	0.96 (0.88-1.05, p=0.403)
	Middle	193135 (99.4)	1141 (0.6)	0.99 (0.91-1.07, p=0.797)	1.03 (0.95-1.13, p=0.443)
	Low	183159 (99.2)	1537 (0.8)	1.41 (1.30-1.52, p<0.001)	1.00 (0.92-1.09, p=0.923)
	Lowest	150184 (99.1)	1412 (0.9)	1.57 (1.46-1.70, p<0.001)	0.93 (0.86-1.02, p=0.126)
	Missing	126613 (99.9)	145 (0.1)	0.19 (0.16-0.23, p<0.001)	0.90 (0.69-1.17, p=0.431)
Myocardial infarction	No	1024244 (99.4)	5846 (0.6)	-	-
	Yes	14187 (96.3)	543 (3.7)	1.48 (1.36-1.62, p<0.001)	1.09 (0.91-1.31, p=0.349)
Congestive heart failure	No	1025290 (99.4)	5696 (0.6)	-	-
	Yes	13141 (95.0)	693 (5.0)	4.86 (4.46-5.28, p<0.001)	0.98 (0.88-1.09, p=0.707)
Peripheral vascular disease	No	1031325 (99.4)	6093 (0.6)	-	-
	Yes	7106 (96.0)	296 (4.0)	9.28 (8.41-10.22, p<0.001)	1.32 (1.18-1.48, p<0.001)
Cerebrovascular disease	No	1015957 (99.4)	5769 (0.6)	-	-
	Yes	22474 (97.3)	620 (2.7)	1.83 (1.70-1.97, p<0.001)	1.21 (1.11-1.33, p<0.001)
Chronic obstructive pulmonary disease	No	1029942 (99.4)	5935 (0.6)	-	-

Dependent: Post-COVID-19 Syndrome	Levels	COVID-positive, No PCS, n (%) N= 1,038,431	COVID-positive, PCS, n (%) N= 6389	COR (95% CI, p-value)	AOR (95% CI, p-value)
	Yes	8489 (94.9)	454 (5.1)	9.49 (8.75-10.28, p<0.001)	1.39 (1.25-1.54, p<0.001)
Chronic other pulmonary disease	No	961200 (99.4)	5570 (0.6)	-	-
	Yes	77231 (99.0)	819 (1.0)	3.71 (3.16-4.31, p<0.001)	1.11 (0.93-1.31, p=0.231)
Rheumatic disease	No	1009276 (99.4)	5836 (0.6)	-	-
	Yes	29155 (98.1)	553 (1.9)	6.37 (5.76-7.02, p<0.001)	1.15 (1.01-1.30, p=0.027)
Dementia	No	1030964 (99.4)	6222 (0.6)	-	-
	Yes	7467 (97.8)	167 (2.2)	3.42 (2.18-5.09, p<0.001)	1.19 (0.74-1.83, p=0.452)
Hemiplegia	No	1032682 (99.4)	6232 (0.6)	-	-
	Yes	5749 (97.3)	157 (2.7)	4.53 (3.84-5.29, p<0.001)	1.19 (0.98-1.42, p=0.069)
Diabetes without chronic complication	No	1037384 (99.4)	6367 (0.6)	-	-
	Yes	1047 (97.9)	22 (2.1)	5.70 (3.48-8.75, p<0.001)	1.46 (0.86-2.32, p=0.136)
Diabetes with chronic complication	No	1026602 (99.4)	5952 (0.6)	-	-
	Yes	11829 (96.4)	437 (3.6)	3.20 (3.00-3.41, p<0.001)	1.11 (1.02-1.22, p=0.021)
Renal disease	No	1029165 (99.4)	5922 (0.6)	-	-
	Yes	9266 (95.2)	467 (4.8)	4.81 (4.10-5.60, p<0.001)	1.41 (1.13-1.77, p=0.003)
Mild liver disease	No	1030789 (99.4)	6270 (0.6)	-	-
	Yes	7642 (98.5)	119 (1.5)	2.56 (2.12-3.06, p<0.001)	1.15 (0.94-1.40, p=0.155)
Ascites	No	1037888 (99.4)	6370 (0.6)	-	-
	Yes	543 (96.6)	19 (3.4)	6.71 (6.13-7.33, p<0.001)	1.12 (1.00-1.25, p=0.042)
Severe liver disease	No	1037628 (99.4)	6357 (0.6)	-	-

Dependent: Post-COVID-19 Syndrome	Levels	COVID-positive, No PCS, n (%) N= 1,038,431	COVID-positive, PCS, n (%) N= 6389	COR (95% CI, p-value)	AOR (95% CI, p-value)
	Yes	803 (96.2)	32 (3.8)	4.30 (3.76-4.88, p<0.001)	1.02 (0.88-1.18, p=0.764)
Peptic ulcer disease	No	1029007 (99.4)	6147 (0.6)	-	-
	Yes	9424 (97.5)	242 (2.5)	7.05 (6.25-7.93, p<0.001)	1.09 (0.95-1.25, p=0.212)
Malignancy	No	972514 (99.5)	5250 (0.5)	-	-
	Yes	65917 (98.3)	1139 (1.7)	8.76 (7.94-9.63, p<0.001)	1.45 (1.28-1.63, p<0.001)
Metastatic solid tumor	No	1032631 (99.4)	6221 (0.6)	-	-
	Yes	5800 (97.2)	168 (2.8)	3.28 (3.00-3.58, p<0.001)	1.22 (1.10-1.35, p<0.001)
HIV/AIDS	No	975108 (99.4)	5828 (0.6)	-	-
	Yes	63323 (99.1)	561 (0.9)	6.50 (4.47-9.10, p<0.001)	1.22 (0.82-1.77, p=0.307)

Supplementary Table 5A. Clinical features of post-COVID-19 syndrome using ICD10 diagnostic codes. Only COVID-positive individuals who received a PCS diagnosis at visits to outpatient or inpatient clinics at least 3 months after COVID-19 are included. The associated diagnostic codes for the same visit where a PCS diagnosis was registered is shown below, and only diagnosis where at least 60 individuals were assigned the code are included. The frequency of individuals that have previously received the ICD-10 diagnosis code for any visit to outpatient and inpatient clinics between 1997 to one month prior to COVID-19 are shown.

**Diagnosis Codes Chapter/ICD 10 codes**	**PCS cohort n = 6 389**		**Women n = 3 308**		**Men n = 3 081**	
	**PCS visit n (%)**	**Previously diagnosed, n (%)**	**PCS visit n (%)**	**Previously diagnosed, n (%)**	**PCS visit n (%)**	**Previously diagnosed, n (%)**
**Infectious diseases**						
B962 (*Escherichia coli* as the cause of diseases)	175 (2.74)	44 (25.14)	96 (2.90)	27 (28.12)	79 (2.56)	17 (21.52)
**Diseases of the blood**						
D509 (anaemia, iron deficiency)	74 (1.16)	26 (35.14)	43 (1.30)	18 (41.86)	31 (1.01)	8 (25.81)
D649 (anaemia)	196 (3.05)	58 (29.74)	93 (2.81)	32 (34.41)	102 (3.31)	26 (25.49)
**Endocrine and metabolic disorders**						
E039 (hypothyroidism)	230 (3.60)	162 (70.43)	176 (5.32)	128 (72.73)	54 (1.75)	34 (62.96)
E118 (DM2, complications unspecified)	93 (1.46)	82 (88.17)	29 (0.88)	28 (96.55)	64 (2.08)	54 (84.38)
E119 (DM2, no complications)	676 (10.58)	504 (74.56)	262 (7.92)	211 (80.53)	414 (13.44)	293 (70.77)
E669 (obesity)	265 (4.15)	117 (44.15)	138 (4.17)	67 (48.55)	127 (4.12)	50 (39.37)
E785 (hyperlipidaemia)	201 (3.15)	93 (46.27)	64 (1.93)	31 (48.44)	137 (4.45)	62 (45.26)
**Mental and cognitive disorders**						
F039 (dementia)	73 (1.14)	42 (57.53)	46 (1.39)	29 (63.04)	27 (0.88)	13 (48.15)
F067 (mild cognitive disorder)	176 (2.75)	24 (13.64)	78 (2.36)	12 (15.38)	98 (3.18)	12 (12.24)
F419 (anxiety disorder)	74 (1.16)	31 (41.89)	36 (1.09)	19 (52.78)	38 (1.23)	12 (31.58)
**Nervous system disorders**						
G409 (epilepsy)	86 (1.35)	70 (81.4)	35 (1.06)	27 (77.14)	51 (1.66)	43 (84.31)

Diagnosis Codes<br>Chapter/ICD 10 codes	PCS cohort n = 6 389		Women n = 3 308		Men n = 3 081	
	PCS visit n (%)	Previously diagnosed, n (%)	PCS visit n (%)	Previously diagnosed, n (%)	PCS visit n (%)	Previously diagnosed, n (%)
G473 (sleep apnoea)	109 (1.71)	78 (71.56)	26 (0.79)	18 (69.23)	83 (2.69)	60 (72.29)
G479 (sleep disorder)	66 (1.03)	4 (6.06)	26 (0.79)	2 (7.69)	40 (1.30)	2 (5)
G819 (hemiplegia)	89 (1.39)	37 (41.57)	40 (1.21)	16 (40)	49 (1.59)	21 (42.86)
G933 (postiviral fatigue syndrome)	164 (2.57)	2 (1.22)	84 (2.54)	1 (1.19)	80 (2.60)	1 (1.25)
**Circulatory system disorders**						
I109 (hypertension)	1539 (24.09)	1120 (72.77)	679 (20.53)	517 (76.14)	860 (27.91)	603 (70.12)
I209 (angina pectoris)	79 (1.24)	55 (69.62)	30 (0.91)	26 (86.67)	49 (1.59)	29 (59.18)
I252 (atherosclerotic heart disease)	313 (4.90)	264 (84.35)	104 (3.14)	85 (81.73)	209 (6.78)	179 (85.65)
I259 (old myocardial infarction)	180 (2.82)	145 (80.56)	51 (1.54)	40 (78.43)	129 (4.19)	105 (81.4)
I269 (pulmonary embolism)	284 (4.45)	24 (8.45)	104 (3.14)	12 (11.54)	180 (5.84)	12 (6.67)
I350 (aortic valve stenosis)	69 (1.08)	48 (69.57)	34 (1.03)	22 (64.71)	35 (1.14)	26 (74.29)
I480 (paroxysmal atrial fibrillation)	165 (2.58)	107 (64.85)	70 (2.12)	51 (72.86)	95 (3.08)	56 (58.95)
I482 (chronic atrial fibrillation)	96 (1.50)	85 (88.54)	35 (1.06)	29 (82.86)	61 (1.98)	56 (91.8)
I489 (atrial fibrillation and flutter)	581 (9.09)	406 (69.88)	267 (8.07)	189 (70.79)	314 (10.19)	217 (69.11)
I509 (heart failure)	554 (8.67)	347 (62.64)	273 (8.25)	169 (61.9)	281 (9.12)	178 (63.35)
I693 (cerebral infarction)	124 (1.94)	91 (73.39)	47 (1.42)	39 (82.98)	77 (2.50)	52 (67.53)
**Respiratory system disorders**						
J159 (bacterial pneumonia)	108 (1.69)	44 (40.74)	44 (1.33)	15 (34.09)	64 (2.08)	29 (45.31)
J189 (pneumonia)	113 (1.77)	40 (35.4)	49 (1.48)	15 (30.61)	64 (2.08)	25 (39.06)
J441 (COPD with acute exacerbation)	87 (1.36)	71 (81.61)	51 (1.54)	39 (76.47)	36 (1.17)	32 (88.89)
J449 (COPD)	343 (5.37)	267 (77.84)	177 (5.35)	139 (78.53)	166 (5.39)	128 (77.11)
J459 (asthma)	349 (5.46)	209 (59.89)	203 (6.14)	126 (62.07)	146 (4.74)	83 (56.85)
J841 (other interstitial pulmonary disease with fibrosis)	74 (1.16)	24 (32.43)	25 (0.76)	10 (40)	49 (1.59)	14 (28.57)

Diagnosis Codes Chapter/ICD 10 codes	PCS cohort n = 6 389		Women n = 3 308		Men n = 3 081	
	PCS visit n (%)	Previously diagnosed, n (%)	PCS visit n (%)	Previously diagnosed, n (%)	PCS visit n (%)	Previously diagnosed, n (%)
J909 (pleural effusion)	144 (2.25)	15 (10.42)	65 (1.96)	5 (7.69)	79 (2.56)	10 (12.66)
J969 (respiratory failure)	89 (1.39)	12 (13.48)	33 (1.00)	7 (21.21)	56 (1.82)	5 (8.93)
**Renal disorders**						
N109 (acute tubulo-interstitial nephritis)	88 (1.38)	22 (25)	36 (1.09)	11 (30.56)	52 (1.69)	11 (21.15)
N179 (acute renal failure)	127 (1.99)	23 (18.11)	42 (1.27)	10 (23.81)	85 (2.76)	13 (15.29)
N183 (CKD, stage 3)	92 (1.44)	47 (51.09)	26 (0.79)	14 (53.85)	66 (2.14)	33 (50)
N184 (CKD, stage 4)	85 (1.33)	74 (87.06)	39 (1.18)	35 (89.74)	46 (1.49)	39 (84.78)
N185 (CKD, stage 5)	67 (1.05)	66 (98.51)	25 (0.76)	25 (100)	42 (1.36)	41 (97.62)
N189 (CKD)	156 (2.44)	98 (62.82)	49 (1.48)	31 (63.27)	107 (3.47)	67 (62.62)
N390 (urinary tract infection)	224 (3.51)	115 (51.34)	110 (3.33)	64 (58.18)	114 (3.70)	51 (44.74)
N409 (prostate hyperplasia)	76 (1.19)	55 (72.37)	0 (0.00)	0 (0)	76 (2.47)	55 (72.37)
**Pregnancy and childbirth**						
O701 (second degree perineal laceration during delivery)	90 (1.41)	20 (22.22)	90 (2.72)	20 (22.22)	NA	NA
O800A (spontaneous vertex delivery)	279 (4.37)	142 (50.9)	278 (8.43)	142 (50.9)	NA	NA
**Symptoms, signs and abnormal clinical laboratory findings**						
Circulatory and respiratory system						
R000 (tachycardia)	76 (1.19)	2 (2.63)	47 (1.42)	1 (2.13)	29 (0.94)	1 (3.45)
R002 (palpitations)	90 (1.41)	10 (11.11)	66 (2.00)	7 (10.61)	24 (0.78)	3 (12.5)
R059 (cough)	139 (2.18)	19 (13.67)	56 (1.69)	7 (12.5)	83 (2.69)	12 (14.46)
R060 (dyspnea)	856 (13.40)	166 (19.39)	426 (12.88)	93 (21.83)	430 (13.96)	73 (16.98)
R074 (chest pain)	247 (3.87)	99 (40.08)	115 (3.48)	46 (40)	132 (4.28)	53 (40.15)
Digestive system and abdomen						

Diagnosis Codes Chapter/ICD 10 codes	PCS cohort n = 6 389		Women n = 3 308		Men n = 3 081	
	**PCS visit n (%)**	**Previously diagnosed, n (%)**	**PCS visit n (%)**	**Previously diagnosed, n (%)**	**PCS visit n (%)**	**Previously diagnosed, n (%)**
R104X (abdominal pain)	115 (1.80)	49 (42.61)	72 (2.18)	36 (50)	43 (1.40)	13 (30.23)
R139 (dysphagia)	135 (2.11)	25 (18.52)	43 (1.30)	9 (20.93)	92 (2.99)	16 (17.39)
R202 (skin paresthesia)	81 (1.27)	8 (9.88)	35 (1.06)	6 (17.14)	46 (1.49)	2 (4.35)
Cognition, perception and emotional state and behaviours						
R418A (subjective mild cognitive dysfunction)	89 (1.39)	1 (1.12)	43 (1.39)	0 (0)	43 (1.40)	1 (2.33)
R429 (dizziness)	110 (1.72)	26 (23.64)	63 (1.90)	13 (20.63)	47 (1.53)	13 (27.66)
General symptoms and signs						
R509 (fever)	172 (2.69)	37 (21.51)	90 (2.72)	19 (21.11)	82 (2.66)	18 (21.95)
R519 (headache)	132 (2.07)	33 (25)	75 (2.27)	19 (25.33)	57 (1.85)	14 (24.56)
R529 (pain)	99 (1.55)	12 (12.12)	39 (1.18)	5 (12.82)	60 (1.95)	7 (11.67)
R539 (malaise and fatigue)	513 (8.03)	38 (7.41)	263 (7.95)	23 (8.75)	250 (8.11)	15 (6)
Abnormal findings on pulmonary diagnostic imaging and functions studies						
R919 (abnormal findings on pulmonary diagnostic imaging)	272 (4.26)	12 (4.41)	80 (2.42)	6 (7.5)	192 (6.23)	6 (3.12)
R942 (abnormal results of pulmonary function studies)	100 (1.57)	2 (2)	21 (0.63)	0 (0)	79 (2.56)	2 (2.53)
**Codes for special purposes**						
U071 (COVID-19, virus identified)	144 (2.25)	NA	50 (1.51)	NA	94 (3.05)	NA

Supplementary Table 5B. Clinical features of post-COVID-19 syndrome using ICD10 diagnostic codes. Only COVID-positive individuals who received a PCS diagnosis at visits to outpatient or inpatient clinics at least 3 months after COVID-19 are included. The associated diagnostic codes for the same visit where a PCS diagnosis was registered is shown below, and only diagnosis where at least 60 individuals were assigned the code are included. The frequency of individuals that have previously received the ICD-10 diagnosis code for any visit to outpatient and inpatient clinics between 1997 to one month prior to COVID-19 are shown. Diagnoses codes are sorted by proportion of individuals who previously received diagnoses codes from smallest to largest proportion.

**Diagnosis Codes**	**PCS cohort n = 6 389**		**Women n = 3 308**		**Men n = 3 081**	
**Chapter/ICD 10 codes**	**PCS visit n (%)**	**Previously diagnosed, n (%)**	**PCS visit n (%)**	**Previously diagnosed, n (%)**	**PCS visit n (%)**	**Previously diagnosed, n (%)**
R418A (subjective mild cognitive disorder)	89 (0.7)	1 (1.12)	43 (1.39)	0 (0)	43 (1.40)	1 (2.33)
G933 (postiviral fatigue syndrome)	164 (1.29)	2 (1.22)	84 (2.54)	1 (1.19)	80 (2.60)	1 (1.25)
R942 (abnormal results of pulmonary function studies)	100 (0.79)	2 (2)	21 (0.63)	0 (0)	79 (2.56)	2 (2.53)
R000 (tachycardia)	76 (0.6)	2 (2.63)	47 (1.42)	1 (2.13)	29 (0.94)	1 (3.45)
R919 (abnormal findings on pulmonary diagnostic imaging)	272 (2.14)	12 (4.41)	80 (2.42)	6 (7.5)	192 (6.23)	6 (3.12)
G479 (sleep disorder)	66 (0.52)	4 (6.06)	26 (0.79)	2 (7.69)	40 (1.30)	2 (5)
R539 (malaise and fatigue)	513 (4.04)	38 (7.41)	263 (7.95)	23 (8.75)	250 (8.11)	15 (6)
I269 (pulmonary embolism)	284 (2.24)	24 (8.45)	104 (3.14)	12 (11.54)	180 (5.84)	12 (6.67)
R202 (skin paresthesia)	81 (0.64)	8 (9.88)	35 (1.06)	6 (17.14)	46 (1.49)	2 (4.35)
J909 (pleural effusion)	144 (1.13)	15 (10.42)	65 (1.96)	5 (7.69)	79 (2.56)	10 (12.66)
R002 (palpitations)	90 (0.71)	10 (11.11)	66 (2.00)	7 (10.61)	24 (0.78)	3 (12.5)
R529 (pain)	99 (0.78)	12 (12.12)	39 (1.18)	5 (12.82)	60 (1.95)	7 (11.67)
J969 (respiratory failure)	89 (0.7)	12 (13.48)	33 (1.00)	7 (21.21)	56 (1.82)	5 (8.93)
F067 (mild cognitive disorder)	176 (1.39)	24 (13.64)	78 (2.36)	12 (15.38)	98 (3.18)	12 (12.24)
R059 (cough)	139 (1.1)	19 (13.67)	56 (1.69)	7 (12.5)	83 (2.69)	12 (14.46)
N179 (acute renal failure)	127 (1)	23 (18.11)	42 (1.27)	10 (23.81)	85 (2.76)	13 (15.29)

Diagnosis Codes<br><br>Chapter/ICD 10 codes	PCS cohort n = 6 389		Women n = 3 308		Men n = 3 081	
	PCS visit n (%)	Previously diagnosed,<br><br>n (%)	PCS visit n (%)	Previously diagnosed,<br><br>n (%)	PCS visit n (%)	Previously diagnosed,<br><br>n (%)
R139 (dysphagia)	135 (1.06)	25 (18.52)	43 (1.30)	9 (20.93)	92 (2.99)	16 (17.39)
R060 (dyspnea)	856 (6.74)	166 (19.39)	426 (12.88)	93 (21.83)	430 (13.96)	73 (16.98)
R509 (fever)	172 (1.36)	37 (21.51)	90 (2.72)	19 (21.11)	82 (2.66)	18 (21.95)
O701 (second degree perineal laceration during delivery)	90 (0.71)	20 (22.22)	90 (2.72)	20 (22.22)	0 (0)	0 (NaN)
R429 (dizziness)	110 (0.87)	26 (23.64)	63 (1.90)	13 (20.63)	47 (1.53)	13 (27.66)
N109 (acute tubulo-interstitial nephritis)	88 (0.69)	22 (25)	36 (1.09)	11 (30.56)	52 (1.69)	11 (21.15)
R519 (headache)	132 (1.04)	33 (25)	75 (2.27)	19 (25.33)	57 (1.85)	14 (24.56)
B962 (*Escherichia coli* as the cause of diseases)	175 (1.38)	44 (25.14)	96 (2.90)	27 (28.12)	79 (2.56)	17 (21.52)
D649 (anaemia)	195 (1.54)	58 (29.74)	93 (2.81)	32 (34.41)	102 (3.31)	26 (25.49)
J841 (other interstitial pulmonary disease with fibrosis)	74 (0.58)	24 (32.43)	25 (0.76)	10 (40)	49 (1.59)	14 (28.57)
D509 (anaemia, iron deficiency)	74 (0.58)	26 (35.14)	43 (1.30)	18 (41.86)	31 (1.01)	8 (25.81)
J189 (pneumonia)	113 (0.89)	40 (35.4)	49 (1.48)	15 (30.61)	64 (2.08)	25 (39.06)
R074 (chest pain)	247 (1.95)	99 (40.08)	115 (3.48)	46 (40)	132 (4.28)	53 (40.15)
J159 (bacterial pneumonia)	108 (0.85)	44 (40.74)	44 (1.33)	15 (34.09)	64 (2.08)	29 (45.31)
G819 (hemiplegia)	89 (0.7)	37 (41.57)	40 (1.21)	16 (40)	49 (1.59)	21 (42.86)
F419 (anxiety disorder)	74 (0.58)	31 (41.89)	36 (1.09)	19 (52.78)	38 (1.23)	12 (31.58)
R104X (abdominal pain)	115 (0.91)	49 (42.61)	72 (2.18)	36 (50)	43 (1.40)	13 (30.23)
E669 (obesity)	265 (2.09)	117 (44.15)	138 (4.17)	67 (48.55)	127 (4.12)	50 (39.37)
E785 (hyperlipidaemia)	201 (1.58)	93 (46.27)	64 (1.93)	31 (48.44)	137 (4.45)	62 (45.26)
O800A (spontaneous vertex delivery)	279 (2.2)	142 (50.9)	278 (8.43)	142 (50.9)	0 (0)	0 (NaN)
N183 (CKD, stage 3)	92 (0.72)	47 (51.09)	26 (0.79)	14 (53.85)	66 (2.14)	33 (50)
N390 (urinary tract infection)	224 (1.77)	115 (51.34)	110 (3.33)	64 (58.18)	114 (3.70)	51 (44.74)

Diagnosis Codes<br><br>Chapter/ICD 10 codes	PCS cohort n = 6 389		Women n = 3 308		Men n = 3 081	
	PCS visit n (%)	Previously diagnosed,<br><br>n (%)	PCS visit n (%)	Previously diagnosed,<br><br>n (%)	PCS visit n (%)	Previously diagnosed,<br><br>n (%)
F039 (dementia)	73 (0.58)	42 (57.53)	46 (1.39)	29 (63.04)	27 (0.88)	13 (48.15)
J459 (asthma)	349 (2.75)	209 (59.89)	203 (6.14)	126 (62.07)	146 (4.74)	83 (56.85)
I509 (heart failure)	554 (4.37)	347 (62.64)	273 (8.25)	169 (61.9)	281 (9.12)	178 (63.35)
N189 (CKD)	156 (1.23)	98 (62.82)	49 (1.48)	31 (63.27)	107 (3.47)	67 (62.62)
I480 (paroxysmal atrial fibrillation)	165 (1.3)	107 (64.85)	70 (2.12)	51 (72.86)	95 (3.08)	56 (58.95)
I350 (aortic valve stenosis)	69 (0.54)	48 (69.57)	34 (1.03)	22 (64.71)	35 (1.14)	26 (74.29)
I209 (angina pectoris)	79 (0.62)	55 (69.62)	30 (0.91)	26 (86.67)	49 (1.59)	29 (59.18)
I489 (atrial fibrillation and flutter)	581 (4.58)	406 (69.88)	267 (8.07)	189 (70.79)	314 (10.19)	217 (69.11)
E039 (hypothyroidism)	230 (1.81)	162 (70.43)	176 (5.32)	128 (72.73)	54 (1.75)	34 (62.96)
G473 (sleep apnoea)	109 (0.86)	78 (71.56)	26 (0.79)	18 (69.23)	83 (2.69)	60 (72.29)
N409 (prostate hyperplasia)	76 (0.6)	55 (72.37)	0 (0.00)	0 (NaN)	76 (2.47)	55 (72.37)
I109 (hypertension)	1539 (12.13)	1120 (72.77)	679 (20.53)	517 (76.14)	860 (27.91)	603 (70.12)
I693 (cerebral infarction)	124 (0.98)	91 (73.39)	47 (1.42)	39 (82.98)	77 (2.50)	52 (67.53)
E119 (DM2, no complications)	676 (5.33)	504 (74.56)	262 (7.92)	211 (80.53)	414 (13.44)	293 (70.77)
J449 (COPD)	343 (2.7)	267 (77.84)	177 (5.35)	139 (78.53)	166 (5.39)	128 (77.11)
I259 (old myocardial infarction)	180 (1.42)	145 (80.56)	51 (1.54)	40 (78.43)	129 (4.19)	105 (81.4)
G409 (epilepsy)	86 (0.68)	70 (81.4)	35 (1.06)	27 (77.14)	51 (1.66)	43 (84.31)
J441 (COPD with acute exacerbation)	87 (0.69)	71 (81.61)	51 (1.54)	39 (76.47)	36 (1.17)	32 (88.89)
I252 (atherosclerotic heart disease)	313 (2.47)	264 (84.35)	104 (3.14)	85 (81.73)	209 (6.78)	179 (85.65)
N184 (CKD, stage 4)	85 (0.67)	74 (87.06)	39 (1.18)	35 (89.74)	46 (1.49)	39 (84.78)
E118 (DM2, complications unspecified)	93 (0.73)	82 (88.17)	29 (0.88)	28 (96.55)	64 (2.08)	54 (84.38)
I482 (chronic atrial fibrillation)	96 (0.76)	85 (88.54)	35 (1.06)	29 (82.86)	61 (1.98)	56 (91.8)

Diagnosis Codes<br><br>Chapter/ICD 10 codes	PCS cohort n = 6 389		Women n = 3 308		Men n = 3 081	
	PCS visit n (%)	Previously diagnosed,<br><br>n (%)	PCS visit n (%)	Previously diagnosed,<br><br>n (%)	PCS visit n (%)	Previously diagnosed,<br><br>n (%)
N185 (CKD, stage 5)	67 (0.53)	66 (98.51)	25 (0.76)	25 (100)	42 (1.36)	41 (97.62)

Supplementary Table 5C. Clinical features of post-COVID-19 syndrome using ICD10 diagnostic codes. Only COVID-positive individuals who received a PCS diagnosis at visits to outpatient or inpatient clinics at least 3 months after COVID-19 are included. The associated diagnostic codes for the same visit where a PCS diagnosis was registered is shown below, and only diagnosis where at least 60 individuals were assigned the code are included. The frequency of individuals that have previously received the ICD-10 diagnosis code for any visit to outpatient and inpatient clinics between 1997 to one month prior to COVID-19 are shown. Stratified by severity of COVID-19.

**Diagnosis Codes**	**No health care N = 2 138**		**Outpatient clinic N = 377**		**Hospitalized N = 2 212**		**Non-invasive ventilation/High-Flow Oxygen N = 471**		**Intensive care unit N = 104**		**Mechanical ventilation N = 1 087**	
	PCS visit n (% of no healthcare)	Previously diagnosed n (% with diagnosis)	PCS visit n (% of out-patient clinics)	Previously diagnosed n (% with diagnosis)	PCS visit n (% of hospitalized)	Previously diagnosed n (% with diagnosis)	PCS visit n (% of NIV/HFO)	Previously diagnosed n (% with diagnosis)	PCS visit n (% of ICU)	Previously diagnosed n (% with diagnosis)	PCS visit n (% of MV)	Previously diagnosed n (% with diagnosis)
**Infectious diseases**												
B962 (*Escherichia coli* as the cause of diseases)	35 (1.64)	9 (25.71)	10 (2.66)	3 (30)	90 (4.07)	24 (26.67)	15 (3.18)	5 (33.33)	5 (4.81)	1 (20)	20 (1.84)	2 (10)
**Diseases of the blood**												
D509 (anaemia, iron deficiency)	20 (0.94)	6 (30)	5 (1.32)	1 (20)	37 (1.67)	16 (43.24)	3 (0.64)	1 (33.33)	0 (0)	0 (0)	9 (0.83)	2 (22.22)
D649 (anaemia)	45 (2.10)	8 (17.78)	6 (1.59)	3 (50)	101 (4.57)	38 (37.62)	14 (2.97)	5 (35.71)	6 (5.77)	1 (16.67)	23 (2.12)	3 (13.04)
**Endocrine and metabolic disorders**												
E039 (hypothyroidism)	58 (2.71)	38 (65.52)	18 (4.77)	11 (61.11)	109 (4.93)	85 (77.98)	19 (4.03)	11 (57.89)	6 (5.77)	4 (66.67)	20 (1.84)	13 (65)
E118 (DM2, complications unspecified)	14 (0.65)	14 (100)	<3	<3	47 (2.12)	43 (91.49)	9 (1.91)	8 (88.89)	3 (2.88)	3 (100)	19 (1.75)	13 (68.42)
E119 (DM2, no complications)	118 (5.52)	92 (77.97)	7 (1.89)	4 (57.14)	328 (14.83)	265 (80.79)	54 (11.46)	44 (81.48)	33 (31.73)	24 (72.73)	136 (12.51)	75 (55.15)
E669 (obesity)	68 (3.2)	27 (39.71)	15 (3.98)	3 (20)	67 (3.03)	44 (65.67)	22 (4.67)	11 (50)	19 (18.27)	10 (52.63)	74 (6.81)	22 (29.73)
E785 (hyperlipidaemia)	52 (2.43)	23 (44.23)	7 (1.86)	2 (28.57)	93 (4.20)	47 (50.54)	14 (2.97)	5 (35.71)	4 (3.85)	4 (100)	31 (2.85)	12 (38.71)

Diagnosis Codes	No health care N = 2 138		Outpatient clinic N = 377		Hospitalized N = 2 212		Non-invasive ventilation/High-Flow Oxygen N = 471		Intensive care unit N = 104		Mechanical ventilation N = 1 087	
	PCS visit n (% of no healthcare)	Previously diagnosed n (% with diagnosis)	PCS visit n (% of out-patient clinics)	Previously diagnosed n (% with diagnosis)	PCS visit n (% of hospitalized)	Previously diagnosed n (% with diagnosis)	PCS visit n (% of NIV/HFO)	Previously diagnosed n (% with diagnosis)	PCS visit n (% of ICU)	Previously diagnosed n (% with diagnosis)	PCS visit n (% of MV)	Previously diagnosed n (% with diagnosis)
**Mental and cognitive disorders**												
F039 (dementia)	28 (1.31)	20 (71.43)	3 (0.80)	2 (66.67)	40 (1.81)	18 (45)	<3	<3	0 (0)	0 (0)	0 (0)	0 (0)
F067 (mild cognitive disorder)	30 (1.40)	7 (23.33)	5 (1.33)	0 (0)	71 (3.21)	15 (21.13)	11 (2.34)	1 (9.09)	7 (6.73)	0 (0)	52 (4.78)	1 (1.92)
F419 (anxiety disorder)	20 (0.94)	9 (45)	4 (1.06)	0 (0)	25 (1.13)	12 (48)	6 (1.27)	4 (66.67)	<3	<3	17 (1.56)	4 (23.53)
**Nervous system disorders**												
G409 (epilepsy)	22 (1.03)	17 (77.27)	4 (1.06)	4 (100)	45 (2.03)	36 (80)	<3	<3	3 (2.88)	3 (100)	11 (1.01)	9 (81.82)
G473 (sleep apnoea)	20 (0.94)	18 (90)	<3	<3	42 (1.90)	31 (73.81)	15 (3.18)	9 (60)	7 (6.73)	4 (57.14)	24 (2.21)	15 (62.5)
G479 (sleep disorder)	8 (0.37)	2 (25)	6 (1.59)	0 (0)	12 (0.54)	0 (0)	6 (1.27)	0 (0)	5 (4.81)	1 (20)	29 (2.67)	1 (3.45)
G819 (hemiplegia)	17 (0.80)	6 (35.29)	<3	<3	48 (2.17)	26 (54.17)	6 (1.27)	3 (50)	<3	0 (0)	16 (1.47)	1 (6.25)
G933 (postiviral fatigue syndrome)	46 (2.2)	2 (4.35)	10 (2.65)	<3	32 (1.45)	0 (0)	22 (4.67)	0 (0)	12 (11.54)	0 (0)	42 (3.86)	0 (0)
**Circulatory system disorders**												
I109 (hypertension)	336 (15.71)	231 (68.75)	37 (9.81)	29 (78.38)	753 (34.04)	608 (80.74)	115 (24.42)	84 (73.04)	55 (52.88)	38 (69.09)	243 (22.36)	130 (53.5)
I209 (angina pectoris)	18 (0.84)	13 (72.22)	3 (0.80)	1 (33.33)	42 (1.90)	31 (73.81)	6 (1.27)	3 (50)	<3	0 (0)	9 (0.83)	7 (77.78)
I252 (atherosclerotic heart disease)	65 (3.04)	58 (89.23)	6 (1.59)	4 (66.67)	191 (8.63)	165 (86.39)	13 (2.76)	9 (69.23)	6 (5.77)	5 (83.33)	32 (2.94)	23 (71.88)
I259 (old myocardial infarction)	24 (1.12)	19 (79.17)	7 (1.86)	5 (71.43)	103 (4.66)	86 (83.5)	18 (3.82)	16 (88.89)	3 (2.88)	3 (100)	25 (2.30)	16 (64)
I269 (pulmonary embolism)	41 (1.92)	3 (7.32)	9 (2.39)	0 (0)	115 (5.20)	14 (12.17)	31 (6.58)	5 (16.13)	16 (15.38)	0 (0)	72 (6.62)	2 (2.78)

Diagnosis Codes	No health care N = 2 138		Outpatient clinic N = 377		Hospitalized N = 2 212		Non-invasive ventilation/High-Flow Oxygen N = 471		Intensive care unit N = 104		Mechanical ventilation N = 1 087	
	PCS visit n (% of no healthcare)	Previously diagnosed n (% with diagnosis)	PCS visit n (% of out-patient clinics)	Previously diagnosed n (% with diagnosis)	PCS visit n (% of hospitalized)	Previously diagnosed n (% with diagnosis)	PCS visit n (% of NIV/HFO)	Previously diagnosed n (% with diagnosis)	PCS visit n (% of ICU)	Previously diagnosed n (% with diagnosis)	PCS visit n (% of MV)	Previously diagnosed n (% with diagnosis)
I350 (aortic valve stenosis)	21 (0.98)	15 (71.43)	<3	0 (0)	34 (1.54)	24 (70.59)	8 (1.70)	6 (75)	<3	<3	3 (0.28)	2 (66.67)
I480 (paroxysmal atrial fibrillation)	31 (1.45)	20 (64.52)	6 (1.59)	5 (83.33)	94 (4.25)	67 (71.28)	6 (1.27)	3 (50)	10 (9.61)	5 (50)	18 (1.66)	7 (38.89)
I482 (chronic atrial fibrillation)	17 (0.80)	14 (82.35)	3 (0.80)	2 (66.67)	67 (3.03)	61 (91.04)	<3	1 (100)	3 (2.88)	2 (66.67)	5 (0.46)	5 (100)
I489 (atrial fibrillation and flutter)	112 (5.24)	85 (75.89)	11 (2.92)	8 (72.73)	360 (16.27)	271 (75.28)	37 (7.86)	20 (54.05)	13 (12.5)	7 (53.85)	48 (4.42)	15 (31.25)
I509 (heart failure)	102 (4.77)	62 (60.78)	7 (1.86)	5 (71.43)	358 (16.18)	234 (65.36)	46 (9.77)	30 (65.22)	10 (9.61)	6 (60)	31 (2.85)	10 (32.26)
I693 (cerebral infarction)	21 (0.98)	19 (90.48)	<3	0 (0)	78 (3.53)	60 (76.92)	8 (1.70)	8 (100)	5 (4.81)	2 (40)	11 (1.01)	2 (18.18)
**Respiratory system disorders**												
J159 (bacterial pneumonia)	22 (1.03)	5 (22.73)	6 (1.59)	3 (50)	54 (2.44)	25 (46.3)	11 (2.34)	7 (63.64)	3 (2.88)	1 (33.33)	12 (1.10)	3 (25)
J189 (pneumonia)	26 (1.22)	8 (30.77)	<3	0 (0)	59 (2.67)	25 (42.37)	13 (2.76)	3 (23.08)	4 (3.85)	3 (75)	10 (0.92)	1 (10)
J441 (COPD with acute exacerbation)	14 (0.65)	9 (64.29)	<3	1 (100)	60 (2.71)	52 (86.67)	9 (1.91)	7 (77.78)	<3	<3	<3	0 (0)
J449 (COPD)	43 (2.01)	39 (90.7)	8 (2.12)	6 (75)	221 (10.00)	181 (81.9)	33 (7)	20 (60.61)	13 (12.5)	9 (69.23)	25 (2.30)	12 (48)
J459 (asthma)	79 (3.70)	41 (51.9)	16 (4.24)	8 (50)	141 (6.38)	104 (73.76)	29 (6.16)	20 (68.97)	21 (20.19)	5 (23.81)	63 (5.80)	31 (49.21)
J841 (other interstitial pulmonary disease with fibrosis)	4 (0.19)	2 (50)	0 (0)	0 (0)	29 (1.31)	13 (44.83)	13 (2.76)	6 (46.15)	4 (3.85)	1 (25)	24 (2.21)	2 (8.33)
J909 (pleural effusion)	30 (1.4)	2 (6.67)	6 (1.59)	0 (0)	79 (3.57)	12 (15.19)	15 (3.18)	1 (6.67)	4 (3.85)	0 (0)	10 (0.92)	0 (0)
J969 (respiratory failure)	5 (0.23)	0 (0)	0 (0)	0 (0)	23 (1.04)	7 (30.43)	12 (2.55)	2 (16.67)	10 (9.61)	0 (0)	39 (3.59)	3 (7.69)
**Renal disorders**												

Diagnosis Codes	No health care N = 2 138		Outpatient clinic N = 377		Hospitalized N = 2 212		Non-invasive ventilation/High-Flow Oxygen N = 471		Intensive care unit N = 104		Mechanical ventilation N = 1 087	
	PCS visit n (% of no healthcare)	Previously diagnosed n (% with diagnosis)	PCS visit n (% of out-patient clinics)	Previously diagnosed n (% with diagnosis)	PCS visit n (% of hospitalized)	Previously diagnosed n (% with diagnosis)	PCS visit n (% of NIV/HFO)	Previously diagnosed n (% with diagnosis)	PCS visit n (% of ICU)	Previously diagnosed n (% with diagnosis)	PCS visit n (% of MV)	Previously diagnosed n (% with diagnosis)
N109 (acute tubulo-interstitial nephritis)	18 (0.85)	3 (16.67)	6 (1.59)	3 (50)	52 (2.35)	13 (25)	5 (1.06)	1 (20)	<3	<3	6 (0.55)	1 (16.67)
N179 (acute renal failure)	18 (0.85)	1 (5.56)	3 (0.80)	0 (0)	63 (2.85)	17 (26.98)	6 (1.27)	3 (50)	5 (4.81)	1 (20)	32 (2.94)	1 (3.12)
N183 (CKD, stage 3)	9 (0.42)	5 (55.56)	<3	<3	39 (1.76)	29 (74.36)	5 (1.06)	4 (80)	5 (4.81)	4 (80)	33 (3.04)	4 (12.12)
N184 (CKD, stage 4)	9 (0.42)	9 (100)	0 (0)	0 (0)	52 (2.35)	49 (94.23)	3 (0.64)	2 (66.67)	5 (4.81)	5 (100)	16 (1.47)	9 (56.25)
N185 (CKD, stage 5)	7 (0.33)	7 (100)	3 (0.80)	3 (100)	34 (1.54)	34 (100)	4 (0.84)	4 (100)	5 (4.81)	5 (100)	14 (1.29)	13 (92.86)
N189 (CKD)	18 (0.84)	10 (55.56)	<3	0 (0)	103 (4.66)	69 (66.99)	15 (3.18)	9 (60)	5 (4.81)	4 (80)	13 (1.20)	6 (46.15)
N390 (urinary tract infection)	46 (2.15)	24 (52.17)	3 (0.80)	2 (66.67)	128 (5.79)	73 (57.03)	19 (4.03)	8 (42.11)	5 (4.81)	1 (20)	23 (2.12)	7 (30.43)
N409 (prostate hyperplasia)	19 (0.89)	15 (78.95)	0 (0)	0 (0)	47 (2.12)	36 (76.6)	4 (0.84)	2 (50)	<3	<3	5 (0.46)	1 (20)
**Pregnancy and childbirth**												
O701 (second degree perineal laceration during delivery)	73 (3.41)	16 (21.92)	11 (2.92)	3 (27.27)	5 (0.23)	1 (20)	<3	0 (0)	0 (0)	0 (0)	0 (0)	0 (0)
O800A (spontaneous vertex delivery)	214 (10)	108 (50.47)	47 (12.47)	24 (51.06)	12 (0.54)	6 (50)	4 (0.84)	2 (50)	<3	<3	<3	<3
**Symptoms, signs and abnormal clinical laboratory findings**												
Circulatory and respiratory system												

Diagnosis Codes	**No health care N = 2 138**		**Outpatient clinic N = 377**		**Hospitalized N = 2 212**		**Non-invasive ventilation/High-Flow Oxygen N = 471**		**Intensive care unit N = 104**		**Mechanical ventilation N = 1 087**	
	PCS visit n (% of no healthcare)	Previously diagnosed n (% with diagnosis)	PCS visit n (% of out-patient clinics)	Previously diagnosed n (% with diagnosis)	PCS visit n (% of hospitalized)	Previously diagnosed n (% with diagnosis)	PCS visit n (% of NIV/HFO)	Previously diagnosed n (% with diagnosis)	PCS visit n (% of ICU)	Previously diagnosed n (% with diagnosis)	PCS visit n (% of MV)	Previously diagnosed n (% with diagnosis)
R000 (tachycardia)	34 (1.59)	1 (2.94)	5 (1.33)	0 (0)	20 (0.90)	1 (5)	3 (0.64)	0 (0)	<3	0 (0)	12 (1.10)	0 (0)
R002 (palpitations)	43 (2.01)	5 (11.63)	12 (3.18)	1 (8.33)	21 (0.95)	2 (9.52)	<3	0 (0)	3 (2.88)	0 (0)	9 (0.83)	2 (22.22)
R059 (cough)	26 (1.22)	3 (11.54)	7 (1.86)	2 (28.57)	27 (1.22)	5 (18.52)	12 (2.55)	2 (16.67)	9 (8.65)	1 (11.11)	58 (5.34)	6 (10.34)
R060 (dyspnea)	178 (8.33)	24 (13.48)	41 (10.88)	5 (12.2)	281 (12.70)	86 (30.6)	91 (19.32)	19 (20.88)	59 (56.73)	9 (15.25)	206 (18.95)	23 (11.17)
R074 (chest pain)	101 (4.72)	27 (26.73)	20 (5.31)	9 (45)	78 (3.53)	42 (53.85)	17 (3.61)	7 (41.18)	5 (4.81)	3 (60)	26 (2.39)	11 (42.31)
Digestive system and abdomen												
R104X (abdominal pain)	42 (1.96)	14 (33.33)	12 (3.18)	5 (41.67)	42 (1.90)	21 (50)	3 (0.64)	1 (33.33)	<3	<3	14 (1.29)	6 (42.86)
R139 (dysphagia)	25 (1.17)	6 (24)	<3	2 (100)	50 (2.26)	14 (28)	6 (1.27)	1 (16.67)	<3	0 (0)	51 (4.69)	2 (3.92)
R202 (skin paresthesia)	16 (0.75)	1 (6.25)	9 (2.34)	1 (11.11)	13 (0.59)	4 (30.77)	4 (0.84)	1 (25)	3 (2.88)	0 (0)	36 (3.31)	1 (2.78)
Cognition, perception and emotional state and behaviours												
R418A (subjective mild cognitive dysfunction)	35 (1.64)	0 (0)	4 (1.06)	1 (25)	21 (0.95)	0 (0)	10 (2.12)	0 (0)	<3	0 (0)	18 (1.66)	0 (0)
R429 (dizziness)	40 (1.87)	8 (20)	5 (1.33)	0 (0)	33 (1.49)	11 (33.33)	6 (1.27)	3 (50)	<3	0 (0)	25 (2.30)	4 (16)
General symptoms and signs												
R509 (fever)	54 (2.53)	8 (14.81)	10 (2.65)	1 (10)	78 (3.53)	21 (26.92)	9 (1.91)	4 (44.44)	5 (4.81)	1 (20)	16 (1.47)	2 (12.5)
R519 (headache)	52 (2.43)	12 (23.08)	11 (2.92)	1 (9.09)	44 (1.99)	16 (36.36)	7 (1.49)	1 (14.29)	<3	0 (0)	17 (1.56)	3 (17.65)
R529 (pain)	11 (0.51)	1 (9.09)	5 (1.33)	1 (20)	21 (0.95)	3 (14.29)	7 (1.49)	1 (14.29)	5 (4.81)	2 (40)	50 (4.60)	4 (8)
R539 (malaise and fatigue)	121 (5.66)	6 (4.96)	24 (6.37)	2 (8.33)	191 (8.63)	26 (13.61)	33 (7)	1 (3.03)	26 (25)	0 (0)	118 (10.86)	3 (2.54)
Abnormal findings on pulmonary diagnostic imaging												

**Diagnosis Codes**	**No health care N = 2 138**		**Outpatient clinic N = 377**		**Hospitalized N = 2 212**		**Non-invasive ventilation/High-Flow Oxygen N = 471**		**Intensive care unit N = 104**		**Mechanical ventilation N = 1 087**	
	PCS visit n (% of no healthcare)	Previously diagnosed n (% with diagnosis)	PCS visit n (% of out-patient clinics)	Previously diagnosed n (% with diagnosis)	PCS visit n (% of hospitalized)	Previously diagnosed n (% with diagnosis)	PCS visit n (% of NIV/HFO)	Previously diagnosed n (% with diagnosis)	PCS visit n (% of ICU)	Previously diagnosed n (% with diagnosis)	PCS visit n (% of MV)	Previously diagnosed n (% with diagnosis)
and functions studies												
R919 (abnormal findings on pulmonary diagnostic imaging)	21 (0.98)	1 (4.76)	7 (1.86)	1 (14.29)	63 (2.84)	7 (11.11)	32 (6.79)	3 (9.38)	31 (29.81)	0 (0)	118 (10.86)	0 (0)
R942 (abnormal results of pulmonary function studies)	<3	0 (0)	0 (0)	0 (0)	13 (0.59)	1 (7.69)	21 (4.46)	1 (4.76)	13 (12.5)	0 (0)	51 (4.69)	0 (0)
**Codes for special purposes**												
U071 (COVID-19, virus identified)	14 (0.65)	NA	<3	NA	48 (2.17)	NA	12 (2.55)	NA	8 (7.69)	NA	60 (5.52)	NA

Supplementary Table 6A. Logistic regression and false discovery rate analysis (FDR) was performed comparing all ICD10 diagnoses for any visit to outpatient and inpatient clinics at least 3 months after COVID-19 for COVID-positive PCS individuals and index date for matched COVID-negative and COVID-positive control individuals. The control individuals were matched on age, sex and county of residence in Sweden; and also matched on initial COVID-19 disease severity for control COVID-19 patients. This in order to adjust for common prevalent diseases that are present in the background population. Only ICD10 codes where at least 60 individuals in the PCS cohort were given that diagnosis code are included. ICD10 codes from Z-chapter are not included.

**ICD chapter/section ICD10 codes**	**PCS patients N= 6 389**		**COVID-negative controls. N= 23 795**			**COVID-positive controls. N= 25 556**		
	Visit n (%)	Previously diagnosed n (%)	Visit n (%)	Previously diagnosed n (%)	OR (95% CI)*	Visit n (%)	Previously diagnosed n (%)	OR (95% CI)*
**Infectious diseases**								
A419 (sepsis)	81 (1.27)	17 (20.99)	10 (0.04)	1 (10)	33.89 (17.01-67.51)	32 (0.13)	4 (12.5)	10.38 (6.86-15.71)
A499 (bacterial infection)	70 (1.10)	6 (8.57)	18 (0.08)	2 (11.11)	15.33 (9.01-26.07)	35 (0.14)	5 (14.29)	8 (5.33-12)
B956 (*Staphylococcus aureus* as cause of disease)	82 (1.28)	16 (19.51)	22 (0.09)	2 (9.09)	14.58 (9.02-23.58)	47 (0.18)	10 (21.28)	6.98 (4.88-9.99)
B962 (*Escherichia coli* as the cause of diseases)	184 (2.88)	1 (0.54)	35 (0.15)	1 (2.86)	21.65 (14.87-31.53)	88 (0.34)	0 (0)	8.82 (6.8-11.45)
B999 (other infectious diseases)	94 (1.47)	8 (8.51)	16 (0.07)	2 (12.5)	25.56 (14.57-44.84)	59 (0.23)	15 (25.42)	6.71 (4.81-9.36)
**Neoplasms**								
C619 (prostate cancer)	93 (1.46)	78 (83.87)	102 (0.43)	85 (83.33)	3.68 (2.75-4.94)	162 (0.63)	135 (83.33)	2.38 (1.83-3.1)
**Diseases of the blood**								
D509 (anemia, iron deficiency)	100 (1.57)	33 (33)	29 (0.12)	8 (27.59)	13.5 (8.87-20.55)	70 (0.27)	31 (44.29)	5.94 (4.35-8.11)
D649 (anemia)	242 (3.79)	79 (32.64)	82 (0.34)	24 (29.27)	11.89 (9.18-15.4)	186 (0.73)	69 (37.1)	5.49 (4.51-6.69)
**Endocrine and metabolic disorders**								
E039 (hypothyroidism)	248 (3.88)	172 (69.35)	87 (0.37)	64 (73.56)	11.54 (8.97-14.86)	141 (0.55)	102 (72.34)	7.59 (6.12-9.4)
E113 (DM2, ophthalmic complications)	76 (1.19)	70 (92.11)	32 (0.13)	30 (93.75)	9.22 (6.07-14.01)	114 (0.45)	99 (86.84)	2.71 (2.02-3.63)

ICD chapter/section ICD10 codes	PCS patients N= 6 389		COVID-negative controls. N= 23 795			COVID-positive controls. N= 25 556		
	Visit n (%)	Previously diagnosed n (%)	Visit n (%)	Previously diagnosed n (%)	OR (95% CI)*	Visit n (%)	Previously diagnosed n (%)	OR (95% CI)*
E117 (DM2, multiple complications)	79 (1.24)	76 (96.2)	11 (0.05)	10 (90.91)	29.81 (15.43-57.59)	67 (0.26)	66 (98.51)	4.76 (3.43-6.6)
E118 (DM2, unspecified complications)	123 (1.93)	112 (91.06)	30 (0.13)	25 (83.33)	15.95 (10.64-23.92)	86 (0.34)	74 (86.05)	6.11 (4.6-8.13)
E119 (DM2, no complications)	733 (11.47)	546 (74.49)	246 (1.03)	189 (76.83)	13.49 (11.52-15.8)	650 (2.54)	514 (79.08)	5.2 (4.64-5.83)
E669 (obesity)	280 (4.38)	128 (45.71)	54 (0.23)	29 (53.7)	20.8 (15.35-28.19)	139 (0.54)	79 (56.83)	8.95 (7.22-11.08)
E785 (hyperlipidaemia)	229 (3.58)	109 (47.6)	114 (0.48)	60 (52.63)	7.83 (6.23-9.85)	172 (0.67)	103 (59.88)	5.65 (4.61-6.92)
E869 (volume depletion)	80 (1.25)	11 (13.75)	36 (0.15)	5 (13.89)	8.57 (5.76-12.77)	44 (0.17)	4 (9.09)	7.52 (5.17-10.93)
E876 (hypokalaemia)	78 (1.22)	16 (20.51)	22 (0.09)	1 (4.55)	15.37 (9.3-25.41)	36 (0.14)	8 (22.22)	9.05 (6.05-13.55)
**Mental and behavioural disorders**								
F039 (dementia)	91 (1.42)	51 (56.04)	38 (0.16)	18 (47.37)	10.09 (6.8-14.98)	80 (0.31)	46 (57.5)	4.62 (3.41-6.25)
F067 (mild cognitive disorder)	197 (3.08)	34 (17.26)	38 (0.16)	11 (28.95)	20.45 (14.33-29.18)	100 (0.39)	42 (42)	8.31 (6.49-10.64)
F412 (mixed anxiety and depressive disorder)	81 (1.27)	45 (55.56)	23 (0.10)	20 (86.96)	12.81 (8.05-20.38)	61 (0.24)	46 (75.41)	5.43 (3.88-7.59)
F419 (anxiety disorder)	118 (1.85)	51 (43.22)	45 (0.19)	25 (55.56)	9.9 (7-14.01)	90 (0.35)	55 (61.11)	5.32 (4.04-7.01)
**Diseases of the nervous system**								
G309 (alzheimers)	61 (0.95)	44 (72.13)	21 (0.09)	16 (76.19)	11.4 (6.87-18.91)	72 (0.28)	47 (65.28)	3.5 (2.47-4.95)
G409 (epilepsy)	102 (1.60)	78 (76.47)	33 (0.14)	26 (78.79)	11.92 (8.01-17.75)	97 (0.38)	79 (81.44)	4.26 (3.22-5.63)
G473 (sleep apnoea)	137 (2.14)	82 (59.85)	36 (0.15)	18 (50)	16.05 (10.85-23.72)	122 (0.48)	58 (47.54)	4.63 (3.61-5.93)
G479 (sleep disorder)	80 (1.25)	13 (16.25)	18 (0.08)	3 (16.67)	16.26 (9.74-27.14)	30 (0.12)	7 (23.33)	10.96 (7.16-16.76)
G819 (hemiplegia)	90 (1.41)	38 (42.22)	28 (0.12)	9 (32.14)	12.85 (8.29-19.9)	66 (0.26)	38 (57.58)	5.56 (4.04-7.67)
G933 (postviral fatigue disorder)	157 (2.46)	2 (1.27)		-		4 (0.02)	0 (0)	157 (58.2-423.53)
**Diseases of eye and adnexa**								

ICD chapter/section ICD10 codes	PCS patients N= 6 389		COVID-negative controls. N= 23 795			COVID-positive controls. N= 25 556		
	Visit n (%)	Previously diagnosed n (%)	Visit n (%)	Previously diagnosed n (%)	OR (95% CI)*	Visit n (%)	Previously diagnosed n (%)	OR (95% CI)*
H041 (lacrimal gland disorder)	62 (0.97)	20 (32.26)	52 (0.22)	27 (51.92)	4.46 (3.07-6.46)	113 (0.44)	47 (41.59)	2.21 (1.62-3.02)
H259 (senile cataract)	136 (2.13)	64 (47.06)	199 (0.84)	119 (59.8)	2.79 (2.22-3.5)	347 (1.36)	198 (57.06)	1.59 (1.3-1.95)
**Diseases of the circulatory system**								
I109 (hypertension)	1700 (26.61)	1249 (73.47)	728 (3.06)	556 (76.37)	17.16 (15.23-19.34)	1221 (4.78)	1010 (82.72)	9.25 (8.41-10.16)
I209 (angina pectoris)	104 (1.63)	73 (70.19)	47 (0.20)	39 (82.98)	8.59 (6.05-12.2)	76 (0.30)	50 (65.79)	5.57 (4.13-7.5)
I251 (atherosclerotic heart disease)	74 (1.16)	31 (41.89)	29 (0.12)	19 (65.52)	9.48 (6.17-14.58)	77 (0.30)	35 (45.45)	3.87 (2.81-5.33)
I252 (old myocardial infarction)	353 (5.53)	294 (83.29)	129 (0.54)	111 (86.05)	12.48 (10.02-15.55)	248 (0.97)	200 (80.65)	6.32 (5.33-7.5)
I259 (chronic ischaemic heart disease)	221 (3.46)	174 (78.73)	119 (0.50)	103 (86.55)	7.99 (6.3-10.13)	200 (0.78)	167 (83.5)	4.71 (3.86-5.73)
I269 (pulmonary embolism)	299 (4.68)	20 (6.69)	27 (0.11)	6 (22.22)	46.09 (30.4-69.88)	88 (0.34)	10 (11.36)	14.72 (11.5-18.85)
I350 (aortic valve stenosis)	82 (1.28)	58 (70.73)	56 (0.24)	45 (80.36)	5.97 (4.2-8.48)	67 (0.26)	43 (64.18)	4.99 (3.6-6.91)
I480 (paroxysmal atrial fibrillation)	210 (3.29)	135 (64.29)	95 (0.40)	78 (82.11)	8.77 (6.84-11.25)	174 (0.68)	132 (75.86)	5.03 (4.1-6.17)
I482 (chronic atrial fibrillation)	127 (1.99)	113 (88.98)	64 (0.27)	57 (89.06)	8.01 (5.89-10.9)	117 (0.46)	104 (88.89)	4.57 (3.53-5.92)
I489 (atrial fibrillation and flutter)	668 (10.46)	465 (69.61)	292 (1.23)	220 (75.34)	12.26 (10.42-14.41)	502 (1.96)	395 (78.69)	6.52 (5.73-7.41)
I509 (heart failure)	668 (10.46)	418 (62.57)	230 (0.97)	162 (70.43)	16.94 (14.1-20.35)	497 (1.94)	334 (67.2)	7.13 (6.24-8.15)
I639 (cerebral infarction)	75 (1.17)	14 (18.67)	37 (0.16)	9 (24.32)	7.94 (5.33-11.83)	72 (0.28)	23 (31.94)	4.17 (3.02-5.76)
I693 (sequelae of cerebral infarction)	149 (2.33)	102 (68.46)	58 (0.24)	50 (86.21)	10.72 (7.79-14.75)	116 (0.45)	88 (75.86)	5.37 (4.19-6.89)
**Diseases of the respiratory system**								

ICD chapter/section ICD10 codes	PCS patients N= 6 389		COVID-negative controls. N= 23 795			COVID-positive controls. N= 25 556		
	Visit n (%)	Previously diagnosed n (%)	Visit n (%)	Previously diagnosed n (%)	OR (95% CI)*	Visit n (%)	Previously diagnosed n (%)	OR (95% CI)*
J159 (bacterial pneumonia)	116 (1.82)	48 (41.38)	36 (0.15)	16 (44.44)	12.56 (8.6-18.34)	30 (0.12)	13 (43.33)	15.47 (10.35-23.11)
J189 (pneumonia)	152 (2.38)	64 (42.11)	41 (0.17)	18 (43.9)	15.54 (10.8-22.38)	67 (0.26)	31 (46.27)	9.53 (7.1-12.79)
J441 (COPD with acute exacerbation)	110 (1.72)	85 (77.27)	26 (0.11)	20 (76.92)	19.64 (12.31-31.36)	56 (0.22)	51 (91.07)	8.57 (6.13-11.98)
J449 (COPD)	391 (6.12)	298 (76.21)	105 (0.44)	86 (81.9)	16.45 (13.06-20.72)	281 (1.10)	227 (80.78)	6.23 (5.3-7.33)
J459 (asthma)	373 (5.84)	229 (61.39)	58 (0.24)	41 (70.69)	25.64 (19.27-34.12)	188 (0.74)	144 (76.6)	8.48 (7.07-10.16)
J841 (other interstitial pulmonary disease with fibrosis)	78 (1.22)	21 (26.92)	6 (0.03)	4 (66.67)	50.26 (21.9-115.34)	18 (0.07)	4 (22.22)	17.33 (10.38-28.94)
J909 (pleural effusion)	159 (2.49)	23 (14.47)	48 (0.20)	8 (16.67)	12.95 (9.32-17.98)	52 (0.20)	7 (13.46)	12.43 (9.06-17.04)
J960 (acute respiratory failure)	91 (1.42)	12 (13.19)	21 (0.09)	3 (14.29)	17.91 (10.91-29.39)	27 (0.11)	7 (25.93)	13.48 (8.77-20.71)
J961 (chronic respiratory failure)	76 (1.19)	23 (30.26)	6 (0.03)	3 (50)	47.18 (20.53-108.4)	27 (0.11)	17 (62.96)	11.61 (7.43-18.12)
J969 (respiratory failure, unspecified)	84 (1.31)	19 (22.62)	5 (0.02)	1 (20)	62.22 (25.23-153.45)	13 (0.05)	3 (23.08)	25.85 (14.41-46.35)
**Diseases of digestive system**								
K590 (constipation)	105 (1.64)	26 (24.76)	52 (0.22)	13 (25)	7.66 (5.49-10.68)	95 (0.37)	28 (29.47)	4.56 (3.44-6.05)
**Diseases of skin tissue**								
L570 (actinic keratosis)	64 (1.00)	47 (73.44)	126 (0.53)	87 (69.05)	1.91 (1.41-2.59)	157 (0.61)	108 (68.79)	1.65 (1.23-2.21)
**Diseases of musculoskeletal system**								
M353 (polymyalgia rheumatica)	70 (1.10)	50 (71.43)	33 (0.14)	23 (69.7)	8.7 (5.7-13.29)	52 (0.20)	38 (73.08)	5.67 (3.92-8.19)
M549 (dorsalgia)	74 (1.16)	34 (45.95)	41 (0.17)	16 (39.02)	6.76 (4.6-9.95)	63 (0.25)	28 (44.44)	4.74 (3.38-6.65)
M791 (myalgia)	65 (1.02)	26 (40)	14 (0.06)	6 (42.86)	16.85 (9.45-30.05)	47 (0.18)	25 (53.19)	5.61 (3.84-8.19)
M819 (osteoporosis)	80 (1.25)	42 (52.5)	41 (0.17)	25 (60.98)	7.89 (5.36-11.63)	66 (0.26)	36 (54.55)	4.89 (3.53-6.79)
**Renal disorders**								
N109 (acute tubulo-interstitial nephritis)	126 (1.97)	31 (24.6)	31 (0.13)	9 (29.03)	15.55 (10.5-23.05)	78 (0.31)	24 (30.77)	6.58 (4.95-8.75)

ICD chapter/section ICD10 codes	PCS patients N= 6 389		COVID-negative controls. N= 23 795			COVID-positive controls. N= 25 556		
	Visit n (%)	Previously diagnosed n (%)	Visit n (%)	Previously diagnosed n (%)	OR (95% CI)*	Visit n (%)	Previously diagnosed n (%)	OR (95% CI)*
N179 (acute renal failure)	163 (2.55)	31 (19.02)	51 (0.21)	9 (17.65)	12.72 (9.24-17.51)	77 (0.30)	16 (20.78)	9 (6.81-11.9)
N183 (CKD, stage 3)	136 (2.13)	78 (57.35)	51 (0.21)	36 (70.59)	10.28 (7.43-14.22)	127 (0.50)	89 (70.08)	4.42 (3.46-5.66)
N184 (CKD, stage 4)	109 (1.71)	95 (87.16)	39 (0.16)	27 (69.23)	10.82 (7.47-15.66)	83 (0.32)	69 (83.13)	5.33 (4-7.11)
N185 (CKD, stage 5)	85 (1.33)	82 (96.47)	14 (0.06)	14 (100)	23.08 (13.11-40.64)	105 (0.41)	101 (96.19)	3.3 (2.47-4.4)
N189 (CKD, unspecified)	201 (3.15)	129 (64.18)	67 (0.28)	42 (62.69)	12.15 (9.14-16.17)	163 (0.64)	103 (63.19)	5.24 (4.24-6.49)
N390 (urinary tract infection)	311 (4.87)	157 (50.48)	102 (0.43)	44 (43.14)	12.86 (10.16-16.27)	239 (0.94)	124 (51.88)	5.85 (4.89-6.99)
N409 (hyperplasia of prostate)	129 (2.02)	80 (62.02)	110 (0.46)	66 (60)	4.73 (3.64-6.16)	171 (0.67)	103 (60.23)	3.18 (2.51-4.02)
**Pregnancy and childbirth**								
O610A (failed induction of labour, delivery within 48 hours)	64 (1.00)	14 (21.88)	14 (0.06)	1 (7.14)	19.3 (10.4-35.81)	27 (0.11)	5 (18.52)	10.07 (6.34-15.99)
O701 (second degree perineal laceration during delivery)	109 (1.71)	23 (21.1)	30 (0.13)	8 (26.67)	16.37 (10.42-25.71)	25 (0.10)	5 (20)	17.44 (11.29-26.93)
O800A (spontaneous vertex delivery)	317 (4.96)	159 (50.16)	69 (0.29)	32 (46.38)	27.7 (19.84-38.67)	95 (0.37)	48 (50.53)	20.07 (15.19-26.52)
**Symptoms, signs and abnormal clinical laboratory findings**								
Circulatory and respiratory system								
R000 (tachycardia)	88 (1.38)	2 (2.27)	8 (0.03)	2 (25)	39.9 (19.33-82.34)	19 (0.07)	3 (15.79)	18.53 (11.28-30.42)
R002 (palpitations)	122 (1.91)	17 (13.93)	21 (0.09)	9 (42.86)	21.25 (13.23-34.14)	53 (0.21)	14 (26.42)	9.63 (6.93-13.39)
R059 (cough)	159 (2.49)	20 (12.58)	13 (0.05)	1 (7.69)	48.31 (26.85-86.9)	43 (0.17)	4 (9.3)	15.09 (10.74-21.2)
R060 (dyspnea)	1099 (17.20)	261 (23.75)	89 (0.37)	32 (35.96)	54.16 (42.86-68.45)	286 (1.12)	99 (34.62)	18.7 (16.21-21.57)
R074 (chest pain)	437 (6.84)	207 (47.37)	95 (0.40)	42 (44.21)	19.44 (15.31-24.69)	288 (1.13)	148 (51.39)	6.56 (5.62-7.66)

ICD chapter/section ICD10 codes	PCS patients N= 6 389		COVID-negative controls. N= 23 795			COVID-positive controls. N= 25 556		
	Visit n (%)	Previously diagnosed n (%)	Visit n (%)	Previously diagnosed n (%)	OR (95% CI)*	Visit n (%)	Previously diagnosed n (%)	OR (95% CI)*
Digestive system and abdomen								
R104X (abdominal pain)	330 (5.17)	158 (47.88)	93 (0.39)	31 (33.33)	13.74 (10.86-17.37)	404 (1.58)	186 (46.04)	3.47 (2.98-4.03)
R139 (dysphagia)	176 (2.75)	38 (21.59)	49 (0.21)	12 (24.49)	13.64 (9.91-18.78)	110 (0.43)	27 (24.55)	6.7 (5.25-8.54)
Skin tissue								
R202 (paresthesia of skin)	108 (1.69)	14 (12.96)	20 (0.08)	2 (10)	20.27 (12.57-32.69)	26 (0.10)	5 (19.23)	17.19 (11.12-26.56)
Nervous and musculoskeletal system								
R298 (other and unspecified symptoms and signs involving the nervous and musculoskeletal systems	141 (2.21)	7 (4.96)	31 (0.13)	2 (6.45)	19.37 (12.82-29.26)	62 (0.24)	9 (14.52)	9.45 (6.97-12.81)
Urinary system								
R319 (haematuria)	94 (1.47)	26 (27.66)	56 (0.24)	11 (19.64)	6.71 (4.79-9.41)	128 (0.50)	34 (26.56)	3.02 (2.3-3.96)
R339 (Urine retention)	103 (1.61)	32 (31.07)	62 (0.26)	13 (20.97)	6.28 (4.58-8.61)	123 (0.48)	46 (37.4)	3.42 (2.62-4.45)
Cognition, perception, emotional state and behavious								
R410 (disorientation)	82 (1.28)	11 (13.41)	26 (0.11)	1 (3.85)	12.39 (7.91-19.4)	75 (0.29)	7 (9.33)	4.44 (3.24-6.09)
R418A (subjective mild cognitive disorder)	95 (1.49)	1 (1.05)	5 (0.02)	1 (20)	70.03 (28.48-172.21)	12 (0.05)	3 (25)	34.33 (18.39-64.09)
R429 (dizziness)	189 (2.96)	54 (28.57)	82 (0.34)	27 (32.93)	8.85 (6.8-11.52)	152 (0.59)	60 (39.47)	5.08 (4.09-6.3)
General symptoms and signs								
R509 (fever)	384 (6.01)	90 (23.44)	54 (0.23)	4 (7.41)	28.44 (21.23-38.1)	152 (0.59)	46 (30.26)	11.1 (9.12-13.51)
R519 (headache)	186 (2.91)	47 (25.27)	31 (0.13)	10 (32.26)	21.91 (14.97-32.06)	105 (0.41)	36 (34.29)	7.39 (5.79-9.43)
R529 (pain)	153 (2.39)	29 (18.95)	34 (0.14)	8 (23.53)	17.28 (11.73-25.45)	94 (0.37)	22 (23.4)	6.61 (5.1-8.56)
R539 (malaise and fatigue)	636 (9.95)	59 (9.28)	59 (0.25)	11 (18.64)	45.83 (34.45-60.98)	149 (0.58)	31 (20.81)	19.63 (16.22-23.75)

ICD chapter/section ICD10 codes	PCS patients N= 6 389		COVID-negative controls. N= 23 795			COVID-positive controls. N= 25 556		
	Visit n (%)	Previously diagnosed n (%)	Visit n (%)	Previously diagnosed n (%)	OR (95% CI)*	Visit n (%)	Previously diagnosed n (%)	OR (95% CI)*
R559 (syncope and collapse)	81 (1.27)	28 (34.57)	45 (0.19)	17 (37.78)	7.01 (4.84-10.16)	91 (0.36)	24 (26.37)	3.56 (2.64-4.8)
R568X (seizures)	66 (1.03)	21 (31.82)	18 (0.08)	1 (5.56)	13.46 (7.99-22.7)	55 (0.22)	18 (32.73)	4.8 (3.36-6.87)
R651 (systemic inflammatory response syndrome of infectious origin with organ failure)	66 (1.03)	7 (10.61)	14 (0.06)	0 (0)	17.93 (10.07-31.94)	27 (0.11)	1 (3.7)	9.78 (6.25-15.3)
Abnormal findings on diagnostic imaging and function studies, without diagnosis								
R919 (abnormal findings on diagnostic imaging)	309 (4.84)	16 (5.18)	19 (0.08)	6 (31.58)	63.52 (39.48-102.2)	49 (0.19)	12 (24.49)	25.7 (18.96-34.83)
R942 (abnormal results of pulmonary function studies)	90 (1.41)	2 (2.22)		-		4 (0.02)	1 (25)	90 (33.06-245.02)

* All FDR p-values <0.0001, apart from L570 for COVID+ which was 0.0073

DM2 Diabetes type 2; CDK chronic kidney disease

Supplementary Table 6B. Logistic regression and false discovery rate analysis (FDR) was performed comparing all ICD10 diagnoses for any visit to outpatient and inpatient clinics at least 3 months after COVID-19 for COVID-positive PCS individuals and index date for matched COVID-negative and COVID-positive control individuals. The control individuals were matched on age, sex and county of residence in Sweden; and also matched on initial COVID-19 disease severity for control COVID-19 patients. This in order to adjust for common prevalent diseases that are present in the background population. Only ICD10 codes where at least 60 individuals in the PCS cohort were given that diagnosis code are included. ICD10 codes from Z-chapter are not included. The diagnosis codes are sorted by proportion of PCS individuals who previously received that or similar diagnoses codes (small to large).

**ICD chapter/section**<br>**ICD10 codes**	**PCS patients N= 6 389**		**COVID-negative controls. N= 23 795**			**COVID-positive controls. N= 25 556**		
	Visit n (%)	Previously diagnosed n (%)	Visit n (%)	Previously diagnosed n (%)	OR (95% CI)*	Visit n (%)	Previously diagnosed n (%)	OR (95% CI)*
B962 (*Escherichia coli* as the cause of diseases)	184 (2.88)	1 (0.54)	35 (0.15)	1 (2.86)	21.65 (14.87-31.53)	88 (0.34)	0 (0)	8.82 (6.8-11.45)
R418A (subjective mild cognitive disorder)	95 (1.49)	1 (1.05)	5 (0.02)	1 (20)	70.03 (28.48-172.21)	12 (0.05)	3 (25)	34.33 (18.39-64.09)
G933 (postviral fatigue disorder)	157 (2.46)	2 (1.27)		-		4 (0.02)	0 (0)	157 (58.2-423.53)
R942 (abnormal results of pulmonary function studies)	90 (1.41)	2 (2.22)		-		4 (0.02)	1 (25)	90 (33.06-245.02)
R000 (tachycardia)	88 (1.38)	2 (2.27)	8 (0.03)	2 (25)	39.9 (19.33-82.34)	19 (0.07)	3 (15.79)	18.53 (11.28-30.42)
R298 (nervous and musculoskeletal systems signs/symptoms)	141 (2.21)	7 (4.96)	31 (0.13)	2 (6.45)	19.37 (12.82-29.26)	62 (0.24)	9 (14.52)	9.45 (6.97-12.81)
R919 (abnormal findings on diagnostic imaging)	309 (4.84)	16 (5.18)	19 (0.08)	6 (31.58)	63.52 (39.48-102.2)	49 (0.19)	12 (24.49)	25.7 (18.96-34.83)
I269 (pulmonary embolism)	299 (4.68)	20 (6.69)	27 (0.11)	6 (22.22)	46.09 (30.4-69.88)	88 (0.34)	10 (11.36)	14.72 (11.5-18.85)
B999 (other infectious diseases)	94 (1.47)	8 (8.51)	16 (0.07)	2 (12.5)	25.56 (14.57-44.84)	59 (0.23)	15 (25.42)	6.71 (4.81-9.36)
A499 (bacterial infection)	70 (1.10)	6 (8.57)	18 (0.08)	2 (11.11)	15.33 (9.01-26.07)	35 (0.14)	5 (14.29)	8 (5.33-12)
R539 (malaise and fatigue)	636 (9.95)	59 (9.28)	59 (0.25)	11 (18.64)	45.83 (34.45-60.98)	149 (0.58)	31 (20.81)	19.63 (16.22-23.75)

ICD chapter/section<br>ICD10 codes	PCS patients N= 6 389		COVID-negative controls. N= 23 795			COVID-positive controls. N= 25 556		
	Visit n (%)	Previously diagnosed n (%)	Visit n (%)	Previously diagnosed n (%)	OR (95% CI)*	Visit n (%)	Previously diagnosed n (%)	OR (95% CI)*
R651 (systemic inflammatory response syndrome of infectious origin with organ failure)	66 (1.03)	7 (10.61)	14 (0.06)	0 (0)	17.93 (10.07-31.94)	27 (0.11)	1 (3.7)	9.78 (6.25-15.3)
R059 (cough)	159 (2.49)	20 (12.58)	13 (0.05)	1 (7.69)	48.31 (26.85-86.9)	43 (0.17)	4 (9.3)	15.09 (10.74-21.2)
R202 (paresthesia of skin)	108 (1.69)	14 (12.96)	20 (0.08)	2 (10)	20.27 (12.57-32.69)	26 (0.10)	5 (19.23)	17.19 (11.12-26.56)
J960 (acute respiratory failure)	91 (1.42)	12 (13.19)	21 (0.09)	3 (14.29)	17.91 (10.91-29.39)	27 (0.11)	7 (25.93)	13.48 (8.77-20.71)
R410 (disorientation)	82 (1.28)	11 (13.41)	26 (0.11)	1 (3.85)	12.39 (7.91-19.4)	75 (0.29)	7 (9.33)	4.44 (3.24-6.09)
E869 (volume depletion)	80 (1.25)	11 (13.75)	36 (0.15)	5 (13.89)	8.57 (5.76-12.77)	44 (0.17)	4 (9.09)	7.52 (5.17-10.93)
R002 (palpitations)	122 (1.91)	17 (13.93)	21 (0.09)	9 (42.86)	21.25 (13.23-34.14)	53 (0.21)	14 (26.42)	9.63 (6.93-13.39)
J909 (pleural effusion)	159 (2.49)	23 (14.47)	48 (0.20)	8 (16.67)	12.95 (9.32-17.98)	52 (0.20)	7 (13.46)	12.43 (9.06-17.04)
G479 (sleep disorder)	80 (1.25)	13 (16.25)	18 (0.08)	3 (16.67)	16.26 (9.74-27.14)	30 (0.12)	7 (23.33)	10.96 (7.16-16.76)
F067 (mild cognitive disorder)	197 (3.08)	34 (17.26)	38 (0.16)	11 (28.95)	20.45 (14.33-29.18)	100 (0.39)	42 (42)	8.31 (6.49-10.64)
I639 (cerebral infarction)	75 (1.17)	14 (18.67)	37 (0.16)	9 (24.32)	7.94 (5.33-11.83)	72 (0.28)	23 (31.94)	4.17 (3.02-5.76)
R529 (pain)	153 (2.39)	29 (18.95)	34 (0.14)	8 (23.53)	17.28 (11.73-25.45)	94 (0.37)	22 (23.4)	6.61 (5.1-8.56)
N179 (acute renal failure)	163 (2.55)	31 (19.02)	51 (0.21)	9 (17.65)	12.72 (9.24-17.51)	77 (0.30)	16 (20.78)	9 (6.81-11.9)
B956 (*Staphylococcus aureus* as cause of disease)	82 (1.28)	16 (19.51)	22 (0.09)	2 (9.09)	14.58 (9.02-23.58)	47 (0.18)	10 (21.28)	6.98 (4.88-9.99)
E876 (hypokalaemia)	78 (1.22)	16 (20.51)	22 (0.09)	1 (4.55)	15.37 (9.3-25.41)	36 (0.14)	8 (22.22)	9.05 (6.05-13.55)
A419 (sepsis)	81 (1.27)	17 (20.99)	10 (0.04)	1 (10)	33.89 (17.01-67.51)	32 (0.13)	4 (12.5)	10.38 (6.86-15.71)
O701 (second degree perineal laceration during delivery)	109 (1.71)	23 (21.1)	30 (0.13)	8 (26.67)	16.37 (10.42-25.71)	25 (0.10)	5 (20)	17.44 (11.29-26.93)
R139 (dysphagia)	176 (2.75)	38 (21.59)	49 (0.21)	12 (24.49)	13.64 (9.91-18.78)	110 (0.43)	27 (24.55)	6.7 (5.25-8.54)
O610A (failed induction of labour, delivery within 48 hours)	64 (1.00)	14 (21.88)	14 (0.06)	1 (7.14)	19.3 (10.4-35.81)	27 (0.11)	5 (18.52)	10.07 (6.34-15.99)
J969 (respiratory failure, unspecified)	84 (1.31)	19 (22.62)	5 (0.02)	1 (20)	62.22 (25.23-153.45)	13 (0.05)	3 (23.08)	25.85 (14.41-46.35)

ICD chapter/section ICD10 codes	PCS patients N= 6 389		COVID-negative controls. N= 23 795			COVID-positive controls. N= 25 556		
	Visit n (%)	Previously diagnosed n (%)	Visit n (%)	Previously diagnosed n (%)	OR (95% CI)*	Visit n (%)	Previously diagnosed n (%)	OR (95% CI)*
R509 (fever)	384 (6.01)	90 (23.44)	54 (0.23)	4 (7.41)	28.44 (21.23-38.1)	152 (0.59)	46 (30.26)	11.1 (9.12-13.51)
R060 (dyspnea)	1099 (17.20)	261 (23.75)	89 (0.37)	32 (35.96)	54.16 (42.86-68.45)	286 (1.12)	99 (34.62)	18.7 (16.21-21.57)
N109 (acute tubulo-interstitial nephritis)	126 (1.97)	31 (24.6)	31 (0.13)	9 (29.03)	15.55 (10.5-23.05)	78 (0.31)	24 (30.77)	6.58 (4.95-8.75)
K590 (constipation)	105 (1.64)	26 (24.76)	52 (0.22)	13 (25)	7.66 (5.49-10.68)	95 (0.37)	28 (29.47)	4.56 (3.44-6.05)
R519 (headache)	186 (2.91)	47 (25.27)	31 (0.13)	10 (32.26)	21.91 (14.97-32.06)	105 (0.41)	36 (34.29)	7.39 (5.79-9.43)
J841 (other interstitial pulmonary disease with fibrosis)	78 (1.22)	21 (26.92)	6 (0.03)	4 (66.67)	50.26 (21.9-115.34)	18 (0.07)	4 (22.22)	17.33 (10.38-28.94)
R319 (haematuria)	94 (1.47)	26 (27.66)	56 (0.24)	11 (19.64)	6.71 (4.79-9.41)	128 (0.50)	34 (26.56)	3.02 (2.3-3.96)
R429 (dizziness)	189 (2.96)	54 (28.57)	82 (0.34)	27 (32.93)	8.85 (6.8-11.52)	152 (0.59)	60 (39.47)	5.08 (4.09-6.3)
J961 (chronic respiratory failure)	76 (1.19)	23 (30.26)	6 (0.03)	3 (50)	47.18 (20.53-108.4)	27 (0.11)	17 (62.96)	11.61 (7.43-18.12)
R339 (Urine retention)	103 (1.61)	32 (31.07)	62 (0.26)	13 (20.97)	6.28 (4.58-8.61)	123 (0.48)	46 (37.4)	3.42 (2.62-4.45)
R568X (seizures)	66 (1.03)	21 (31.82)	18 (0.08)	1 (5.56)	13.46 (7.99-22.7)	55 (0.22)	18 (32.73)	4.8 (3.36-6.87)
H041 (lacrimal gland disorder)	62 (0.97)	20 (32.26)	52 (0.22)	27 (51.92)	4.46 (3.07-6.46)	113 (0.44)	47 (41.59)	2.21 (1.62-3.02)
D649 (anemia)	242 (3.79)	79 (32.64)	82 (0.34)	24 (29.27)	11.89 (9.18-15.4)	186 (0.73)	69 (37.1)	5.49 (4.51-6.69)
D509 (anemia, iron deficiency)	100 (1.57)	33 (33)	29 (0.12)	8 (27.59)	13.5 (8.87-20.55)	70 (0.27)	31 (44.29)	5.94 (4.35-8.11)
R559 (syncope and collapse)	81 (1.27)	28 (34.57)	45 (0.19)	17 (37.78)	7.01 (4.84-10.16)	91 (0.36)	24 (26.37)	3.56 (2.64-4.8)
M791 (myalgia)	65 (1.02)	26 (40)	14 (0.06)	6 (42.86)	16.85 (9.45-30.05)	47 (0.18)	25 (53.19)	5.61 (3.84-8.19)
J159 (bacterial pneumonia)	116 (1.82)	48 (41.38)	36 (0.15)	16 (44.44)	12.56 (8.6-18.34)	30 (0.12)	13 (43.33)	15.47 (10.35-23.11)
I251 (atherosclerotic heart disease)	74 (1.16)	31 (41.89)	29 (0.12)	19 (65.52)	9.48 (6.17-14.58)	77 (0.30)	35 (45.45)	3.87 (2.81-5.33)
J189 (pneumonia)	152 (2.38)	64 (42.11)	41 (0.17)	18 (43.9)	15.54 (10.8-22.38)	67 (0.26)	31 (46.27)	9.53 (7.1-12.79)
G819 (hemiplegia)	90 (1.41)	38 (42.22)	28 (0.12)	9 (32.14)	12.85 (8.29-19.9)	66 (0.26)	38 (57.58)	5.56 (4.04-7.67)
F419 (anxiety disorder)	118 (1.85)	51 (43.22)	45 (0.19)	25 (55.56)	9.9 (7-14.01)	90 (0.35)	55 (61.11)	5.32 (4.04-7.01)

ICD chapter/section<br>ICD10 codes	PCS patients N= 6 389		COVID-negative controls. N= 23 795			COVID-positive controls. N= 25 556		
	Visit n (%)	Previously diagnosed n (%)	Visit n (%)	Previously diagnosed n (%)	OR (95% CI)*	Visit n (%)	Previously diagnosed n (%)	OR (95% CI)*
E669 (obesity)	280 (4.38)	128 (45.71)	54 (0.23)	29 (53.7)	20.8 (15.35-28.19)	139 (0.54)	79 (56.83)	8.95 (7.22-11.08)
M549 (dorsalgia)	74 (1.16)	34 (45.95)	41 (0.17)	16 (39.02)	6.76 (4.6-9.95)	63 (0.25)	28 (44.44)	4.74 (3.38-6.65)
H259 (senile cataract)	136 (2.13)	64 (47.06)	199 (0.84)	119 (59.8)	2.79 (2.22-3.5)	347 (1.36)	198 (57.06)	1.59 (1.3-1.95)
R074 (chest pain)	437 (6.84)	207 (47.37)	95 (0.40)	42 (44.21)	19.44 (15.31-24.69)	288 (1.13)	148 (51.39)	6.56 (5.62-7.66)
E785 (hyperlipidaemia)	229 (3.58)	109 (47.6)	114 (0.48)	60 (52.63)	7.83 (6.23-9.85)	172 (0.67)	103 (59.88)	5.65 (4.61-6.92)
R104X (abdominal pain)	330 (5.17)	158 (47.88)	93 (0.39)	31 (33.33)	13.74 (10.86-17.37)	404 (1.58)	186 (46.04)	3.47 (2.98-4.03)
O800A	317 (4.96)	159 (50.16)	69 (0.29)	32 (46.38)	27.7 (19.84-38.67)	95 (0.37)	48 (50.53)	20.07 (15.19-26.52)
N390 (urinary tract infection)	311 (4.87)	157 (50.48)	102 (0.43)	44 (43.14)	12.86 (10.16-16.27)	239 (0.94)	124 (51.88)	5.85 (4.89-6.99)
M819 (osteoporosis)	80 (1.25)	42 (52.5)	41 (0.17)	25 (60.98)	7.89 (5.36-11.63)	66 (0.26)	36 (54.55)	4.89 (3.53-6.79)
F412 (mixed anxiety and depressive disorder)	81 (1.27)	45 (55.56)	23 (0.10)	20 (86.96)	12.81 (8.05-20.38)	61 (0.24)	46 (75.41)	5.43 (3.88-7.59)
F039 (dementia)	91 (1.42)	51 (56.04)	38 (0.16)	18 (47.37)	10.09 (6.8-14.98)	80 (0.31)	46 (57.5)	4.62 (3.41-6.25)
N183 (CKD, stage 3)	136 (2.13)	78 (57.35)	51 (0.21)	36 (70.59)	10.28 (7.43-14.22)	127 (0.50)	89 (70.08)	4.42 (3.46-5.66)
G473 (sleep apnoea)	137 (2.14)	82 (59.85)	36 (0.15)	18 (50)	16.05 (10.85-23.72)	122 (0.48)	58 (47.54)	4.63 (3.61-5.93)
J459 (asthma)	373 (5.84)	229 (61.39)	58 (0.24)	41 (70.69)	25.64 (19.27-34.12)	188 (0.74)	144 (76.6)	8.48 (7.07-10.16)
N409 (hyperplasia of prostate)	129 (2.02)	80 (62.02)	110 (0.46)	66 (60)	4.73 (3.64-6.16)	171 (0.67)	103 (60.23)	3.18 (2.51-4.02)
I509 (heart failure)	668 (10.46)	418 (62.57)	230 (0.97)	162 (70.43)	16.94 (14.1-20.35)	497 (1.94)	334 (67.2)	7.13 (6.24-8.15)
N189 (CKD, unspecified)	201 (3.15)	129 (64.18)	67 (0.28)	42 (62.69)	12.15 (9.14-16.17)	163 (0.64)	103 (63.19)	5.24 (4.24-6.49)
I480 (paroxysmal atrial fibrillation)	210 (3.29)	135 (64.29)	95 (0.40)	78 (82.11)	8.77 (6.84-11.25)	174 (0.68)	132 (75.86)	5.03 (4.1-6.17)
I693 (sequelae of cerebral infarction)	149 (2.33)	102 (68.46)	58 (0.24)	50 (86.21)	10.72 (7.79-14.75)	116 (0.45)	88 (75.86)	5.37 (4.19-6.89)
E039 (hypothyroidism)	248 (3.88)	172 (69.35)	87 (0.37)	64 (73.56)	11.54 (8.97-14.86)	141 (0.55)	102 (72.34)	7.59 (6.12-9.4)
I489 (atrial fibrillation and flutter)	668 (10.46)	465 (69.61)	292 (1.23)	220 (75.34)	12.26 (10.42-14.41)	502 (1.96)	395 (78.69)	6.52 (5.73-7.41)

ICD chapter/section<br>ICD10 codes	PCS patients N= 6 389		COVID-negative controls. N= 23 795			COVID-positive controls. N= 25 556		
	Visit n (%)	Previously diagnosed n (%)	Visit n (%)	Previously diagnosed n (%)	OR (95% CI)*	Visit n (%)	Previously diagnosed n (%)	OR (95% CI)*
I209 (angina pectoris)	104 (1.63)	73 (70.19)	47 (0.20)	39 (82.98)	8.59 (6.05-12.2)	76 (0.30)	50 (65.79)	5.57 (4.13-7.5)
I350 (aortic valve stenosis)	82 (1.28)	58 (70.73)	56 (0.24)	45 (80.36)	5.97 (4.2-8.48)	67 (0.26)	43 (64.18)	4.99 (3.6-6.91)
M353 (polymyalgia rheumatica)	70 (1.10)	50 (71.43)	33 (0.14)	23 (69.7)	8.7 (5.7-13.29)	52 (0.20)	38 (73.08)	5.67 (3.92-8.19)
G309 (alzheimers)	61 (0.95)	44 (72.13)	21 (0.09)	16 (76.19)	11.4 (6.87-18.91)	72 (0.28)	47 (65.28)	3.5 (2.47-4.95)
L570 (actinic keratosis)	64 (1.00)	47 (73.44)	126 (0.53)	87 (69.05)	1.91 (1.41-2.59)	157 (0.61)	108 (68.79)	1.65 (1.23-2.21)
I109 (hypertension)	1700 (26.61)	1249 (73.47)	728 (3.06)	556 (76.37)	17.16 (15.23-19.34)	1221 (4.78)	1010 (82.72)	9.25 (8.41-10.16)
E119 (DM2, no complications)	733 (11.47)	546 (74.49)	246 (1.03)	189 (76.83)	13.49 (11.52-15.8)	650 (2.54)	514 (79.08)	5.2 (4.64-5.83)
J449 (COPD)	391 (6.12)	298 (76.21)	105 (0.44)	86 (81.9)	16.45 (13.06-20.72)	281 (1.10)	227 (80.78)	6.23 (5.3-7.33)
G409 (epilepsy)	102 (1.60)	78 (76.47)	33 (0.14)	26 (78.79)	11.92 (8.01-17.75)	97 (0.38)	79 (81.44)	4.26 (3.22-5.63)
J441 (COPD with acute exacerbation)	110 (1.72)	85 (77.27)	26 (0.11)	20 (76.92)	19.64 (12.31-31.36)	56 (0.22)	51 (91.07)	8.57 (6.13-11.98)
I259 (chronic ischaemic heart disease)	221 (3.46)	174 (78.73)	119 (0.50)	103 (86.55)	7.99 (6.3-10.13)	200 (0.78)	167 (83.5)	4.71 (3.86-5.73)
I252 (old myocardial infarction)	353 (5.53)	294 (83.29)	129 (0.54)	111 (86.05)	12.48 (10.02-15.55)	248 (0.97)	200 (80.65)	6.32 (5.33-7.5)
C619 (prostate cancer)	93 (1.46)	78 (83.87)	102 (0.43)	85 (83.33)	3.68 (2.75-4.94)	162 (0.63)	135 (83.33)	2.38 (1.83-3.1)
N184 (CKD, stage 4)	109 (1.71)	95 (87.16)	39 (0.16)	27 (69.23)	10.82 (7.47-15.66)	83 (0.32)	69 (83.13)	5.33 (4-7.11)
I482 (chronic atrial fibrillation)	127 (1.99)	113 (88.98)	64 (0.27)	57 (89.06)	8.01 (5.89-10.9)	117 (0.46)	104 (88.89)	4.57 (3.53-5.92)
E118 (DM2, unspecified complications)	123 (1.93)	112 (91.06)	30 (0.13)	25 (83.33)	15.95 (10.64-23.92)	86 (0.34)	74 (86.05)	6.11 (4.6-8.13)
E113 (DM2, ophthalmic complications)	76 (1.19)	70 (92.11)	32 (0.13)	30 (93.75)	9.22 (6.07-14.01)	114 (0.45)	99 (86.84)	2.71 (2.02-3.63)
E117 (DM2, multiple complications)	79 (1.24)	76 (96.2)	11 (0.05)	10 (90.91)	29.81 (15.43-57.59)	67 (0.26)	66 (98.51)	4.76 (3.43-6.6)
N185 (CKD, stage 5)	85 (1.33)	82 (96.47)	14 (0.06)	14 (100)	23.08 (13.11-40.64)	105 (0.41)	101 (96.19)	3.3 (2.47-4.4)
